# Dysregulation of sphingolipid-metabolizing enzymes in Friedreich’s ataxia: *In vitro* and *in vivo* insights into therapeutic targeting

**DOI:** 10.1016/j.isci.2026.116479

**Published:** 2026-06-22

**Authors:** Zenouska Ramchunder, Ester Kalef-Ezra, Saqlain Suleman, Fred Jonathan Edzeamey, Sandor Szunyogh, Owen Gittins, Natalia Castro Mena, Richard Wade-Martins, Adamo Valle, Charareh Pourzand, Sara Anjomani Virmouni

**Affiliations:** 1 Department of Biosciences, College of Health, Medicine and Life Sciences (CHMLS), Brunel University of London, London, UK; 2 Department of Physiology, Anatomy and Genetics, University of Oxford, Oxford, UK; 3 University of Cambridge, Cambridge, UK; 4 Energy Metabolism and Nutrition, Research Institute of Health Sciences (IUNICS), University of the Balearic Islands, Palma, Spain; 5 Health Research Institute of the Balearic Islands (IdISBa), Palma, Spain; 6 Biomedical Research Networking Center for Physiopathology of Obesity and Nutrition (CIBEROBN CB06/03/0043), Instituto de Salud Carlos III, Madrid, Spain; 7 Department of Life Sciences, University of Bath, Bath, UK

**Keywords:** Physiology, Genetics, Molecular biology, Cell biology

## Abstract

Friedreich’s ataxia (FRDA) is an inherited neurodegenerative disorder caused by a GAA repeat expansion within the *FXN* gene, leading to reduced frataxin levels. This deficiency results in mitochondrial dysregulation, oxidative stress, and progressive cell death. Currently, only one approved treatment exists for FRDA in the United States, Canada, and the European Union, which improves neurological outcomes but has not been fully evaluated for broader disease symptoms. Therefore, identifying new therapeutic targets remains essential. Sphingolipids are increasingly recognized for their roles in neurodegeneration with emerging evidence indicating their dysregulation in FRDA. Here, we investigate whether sphingolipid-metabolizing enzymes are similarly affected and assess the therapeutic potential of targeting them. Our findings demonstrate that these enzymes are dysregulated across multiple FRDA models. Importantly, their modulation *in vitro* and *in vivo* significantly reduces mitochondrial dysfunction, enhances frataxin expression, and improves key pathological features of the disease, highlighting sphingolipid metabolism as a promising therapeutic target for FRDA.

## Highlights

• Sphingolipid metabolism is disrupted across multiple FRDA models
• Modulating sphingolipid enzymes restores mitochondrial function
• Targeting sphingolipids increases frataxin expression *in vitro* and *in vivo*
• Sphingolipid pathways are promising therapeutic targets in FRDA

## Graphical abstract

**Figure:**
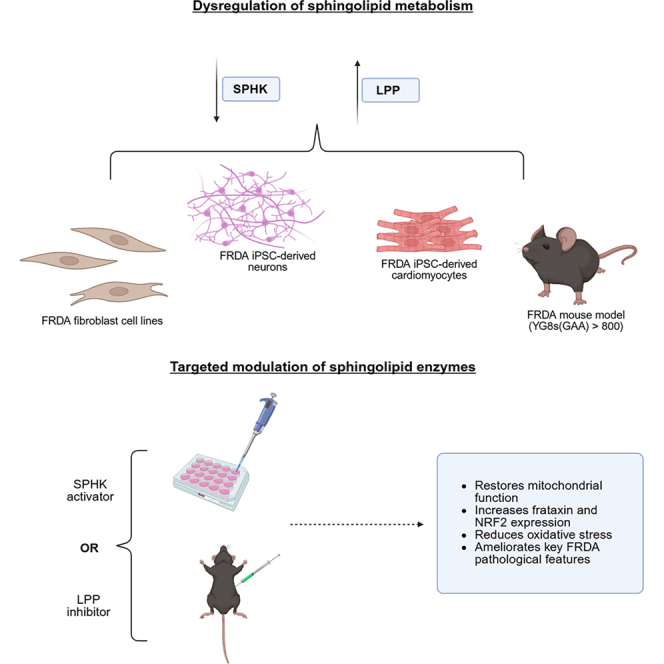

## Introduction

Friedreich’s ataxia (FRDA) is a neurodegenerative autosomal recessive disease^1^ and the most commonly inherited ataxia.^2^ The disease is characterized by a triplet repeat expansion of GAA located on chromosome 9q13 in intron 1 of the frataxin gene, *FXN*.^2^^,^^3^ Among the Western European population, FRDA has been shown to affect approximately 1 in 20,000–1 in 125,000,^4^ with about 1 in 50–1 in 100 being carriers for the disease.^5^^,^^6^ This GAA repeat expansion results in reduced transcription and, therefore, lower levels of the frataxin protein, a mitochondrial protein crucial for the formation of iron-sulfur clusters.^3^^,^^7^ Reduced frataxin protein expression leads to increased iron accumulation, leading to oxidative stress and eventually cell death.^3^^,^^7^ Oxidative stress has been investigated using several models, including *Drosophila*, *Caenorhabditis elegans*, and *Galleria mellonella,*^8^^,^^9^ with some of these models being used in specific FRDA investigations,^10^^,^^11^ as oxidative stress is a hallmark of the disease.

Symptoms of FRDA typically begin to manifest around puberty^2^ with neurological symptoms of the disease typically arising from pathogenesis of the cerebellum and dorsal root ganglia (DRG) neurons.^6^ A study conducted in 1997 found that patients suffering from FRDA exhibited limb and gait ataxia, as well as dysarthria.^12^ Additional symptoms include cardiac hypertrophy and diabetes mellitus.^13^

Currently, only one drug, omaveloxolone (SKYCLARYS), has been approved by the Food and Drug Administration (FDA) in the United States and the European Commission for the treatment of FRDA.^3^^,^^14^^,^^15^ Although omaveloxolone has shown efficacy in improving modified Friedreich's ataxia rating scale (mFARS) in clinical trials—an achievement not realized by previous drugs—its ability to enhance other aspects of the disease, such as skeletal deformities and cardiomyopathy, remains to be determined.^3^ Omaveloxolone acts through the nuclear factor-erythroid 2 p45-related factor 2 (Nrf2), a transcription factor that regulates the expression of antioxidant genes containing antioxidant response elements (AREs) in their promoters, such as glutathione S-transferase and glutathione synthetase.^14^^,^^16^^,^^17^^,^^18^^,^^19^ The efficacy of this drug, specifically targeting Nrf2, underscores the significance of this pathway and oxidative stress in modifying the course of the disease.^18^^,^^19^ Other antioxidants have also been investigated in FRDA, such as vatiquinone, which when evaluated using mFARS in the MOVE-FA phase 3 clinical trial showed significant improvements in patients,^3^ as well as combination therapies of antioxidants in preclinical studies, which have shown increases in *Nrf2* and *FXN in vitro.*^20^

Sphingolipids are bioactive lipids that play a critical role in regulating cellular processes such as survival, apoptosis, and proliferation.^21^^,^^22^ Among the most well-characterized bioactive sphingolipids are ceramide (Cer), ceramide-1-phosphate (C1P), sphingosine (Sph), and sphingosine-1-phosphate (S1P), which influence stress resistance, proliferation, differentiation, and the maturation of nervous system cells.^23^ These molecules have opposing roles in cellular survival signaling, in a dynamic balance known as the “sphingolipid rheostat.”^21^^,^^22^ Cer and Sph promote cell death, while C1P and S1P support cell survival.^21^^,^^22^ Cer is the central molecule in sphingolipid metabolism, synthesized either through the *de novo* biosynthetic pathway, via the hydrolysis of sphingomyelin by sphingomyelinases, or through the “salvage pathway,” which recycles Sph from complex sphingolipids such as gangliosides.^21^^,^^22^^,^^24^ Cer can be converted into Sph, catalyzed by cerimidase (*CDase*), which is phosphorylated by Sph kinase (*SPHK*) to yield S1P.^21^^,^^22^ Cer can also be directly phosphorylated by ceramide kinase (*CERK*) to form C1P and converted back into Cer by lipid phosphatase Case (*LPP*).^21^^,^^22^ *LPP* is also involved in the conversion of S1P to Sph and the degradation of lysophophatidic acid (LPA).^25^ S1P binds to S1P receptors (S1PRs), regulating pathways that promote cell survival and growth, such as the PI3K-Akt pathway.^21^ Similarly, LPA binds to LPA receptors (LPARs), activating the PI3K-Akt pathway.^25^ In contrast, Cer has been shown to activate enzymes that generate reactive oxygen species (ROS), increasing oxidative stress.^26^ Interestingly, Nrf2 has also been shown to be involved in the sphingolipid pathway.^27^^,^^28^ Dysregulation of sphingolipids has been observed in several neurodegenerative disorders such as Alzheimer’s and Huntington’s diseases.^29^

Recently, there have been studies looking at how sphingolipid metabolism may be involved in FRDA. A 2016 study using *fh* mutant fly models found that reduced frataxin levels led to iron toxicity, which was mediated by an increase in *de novo* sphingolipid synthesis, primarily involving dihydroceramide and dihydrosphingosine.^10^ In 2022, another study investigated the metabolomics of FRDA using patient skin biopsies, reporting that 18 different Cer chains were elevated in FRDA, with a correlation between the length of the GAA repeat expansion and the total number of increased Cers.^30^ Given the evidence linking sphingolipid dysregulation to hallmark events of FRDA, such as altered iron metabolism, oxidative stress, and apoptosis, it is plausible that this dysregulation may be associated with changes in the levels of enzymes responsible for converting these lipid species. This study aims to investigate the enzymes involved in the formation of Cer and S1P, the main components of the sphingolipid rheostat, across various FRDA models, with a focus on SPHK1/2 and LPP1/2/3, the key enzymes involved in the conversion of these species. In addition, we explore the impact of targeting these enzymes on mitochondrial dysfunction and frataxin expression. To our knowledge, this is the first study to examine sphingolipid-metabolizing enzymes in FRDA and to target them using either small-molecule drugs or short hairpin RNA (shRNA), both *in vitro* and *in vivo*.

## Results

### Altered expression and activity of the sphingolipid-metabolizing enzymes

**Figure 1:**
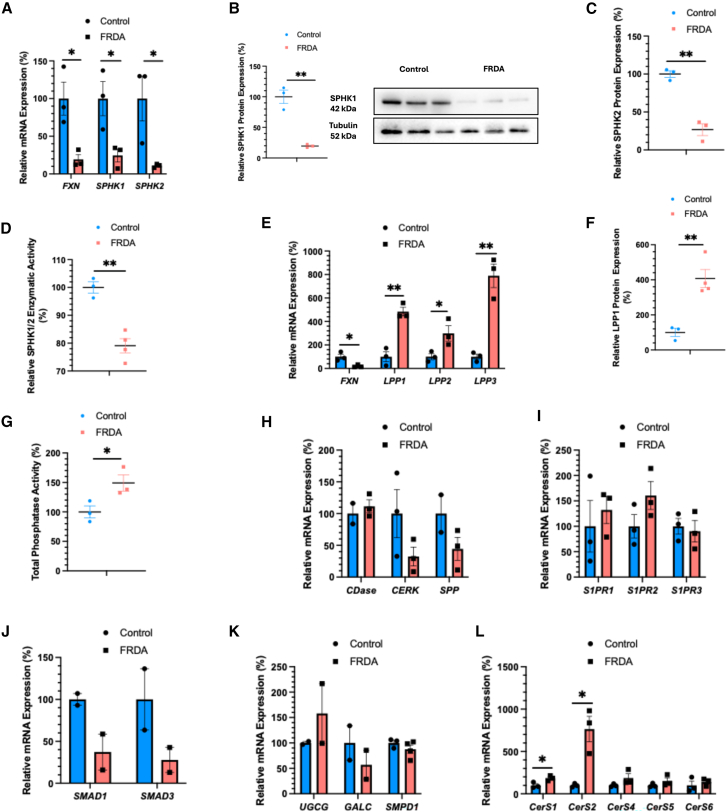
Dysregulation of *SPHK* and *LPP* in human FRDA fibroblast cell lines (A) *SPHK1*/*2* gene expression measured by RT-qPCR, *n* = 3. (B) Representative immunoblots outlining SPHK1 protein expression, *n* = 3, and relative quantification of SPHK1 bands normalized to tubulin and control. (C) SPHK2 protein expression quantified by ELISA, *n* = 3. (D) SPHK1/2 enzymatic activity measured using a kinase activity assay, *n* = 3–4. (E) *LPP1*/*2*/*3* gene expression measured by RT-qPCR, *n* = 3. (F) LPP1 protein expression quantified by ELISA, *n* = 3–4. (G) Total phosphatase activity measured using a malachite assay, *n* = 3. (H–L) RT-qPCR analysis of (H) *CDase*, *CERK*, and *SPP*, *n* = 2–3; (I) *S1PR1*/*2*/*3*, *n* = 3; (J) *SMAD1* and *SMAD3*, *n* = 2; (K) *UGCG*, *GALC*, and *SMPD1*, *n* = 2–4, and (L) *CerS1*/*2*/*4*/*5*/*6*, *n* = 3, gene expression. Data are represented as mean ± SEM. Statistical significance was determined using an unpaired, two-tailed Student’s *t* test (∗*p <* 0.05, ∗∗*p <* 0.01).

To identify if changes in Cer and S1P are related to *SPHK* and *LPP* expression, the expression levels and enzymatic activity of *SPHK* and *LPP* were investigated in human FRDA fibroblast cell lines. The mRNA expression of both *SPHK1* (*p* = 0.0360) and *SPHK2* (*p* = 0.0400) were significantly reduced in human FRDA fibroblast cell lines (Figure 1A). This decrease in mRNA expression was then followed up using western blot and ELISA to detect SPHK1 and SPHK2 protein expression in these cell lines. Western blot showed a significant reduction in SPHK1 protein expression (Figure 1B, *p* = 0.0018), while ELISA revealed a significant reduction in SPHK2 protein expression (Figure 1C, *p* = 0.0012). Enzymatic activity assays for SPHK1/2 showed a trend toward reduced activity (Figure 1D, *p* = 0.0018), indicating a decrease in both expression and activity of SPHK in human FRDA fibroblast cell lines.

Similar experiments were performed to examine potential changes in LPP expression. The mRNA levels of *LPP1* (*p* = 0.0025), *LPP2* (*p* = 0.0471), and *LPP3* (*p* = 0.0026) were all significantly elevated in human FRDA fibroblast cell lines compared to controls (Figure 1E). ELISA confirmed a significant increase in *LPP1* protein expression in these cell lines (Figure 1F, *p* = 0.0049). In addition, a total phosphatase assay revealed a significant increase in overall phosphatase activity in human FRDA fibroblast cell lines (Figure 1G, *p* = 0.0460).

*SPHK* and *LPP* were the two main enzymes focused on due to their direct involvement in S1P and Cer expression levels. However, other enzymes also regulate S1P and Cer through alternative pathways. Therefore, mRNA expression data were also collected for other sphingolipid-metabolizing enzymes from the *de novo* and *salvage* sphingolipid synthesis pathways. No significant changes were found in the levels of *CDase*, *CERK*, or *SPP*, or in *S1PR1/2/3* (Figures 1H and 1I). Similarly, no significant changes were detected in *SMAD1*, *SMAD3*, *GALC*, *SMPD1*, and U*GCG* (Figures 1J and 1K). However, significant increases were found in *CerS1* (*p* = 0.0310) and *CerS2* (*p* = 0.0116), but not in the other *CerS* isoforms in human FRDA fibroblast cell lines (Figure 1L). These data suggest that the alterations in sphingolipids observed by other groups may be primarily driven by changes in *SPHK* and *LPP*, as these enzymes influence the rate of sphingolipid formation.

To determine whether changes in sphingolipid-metabolizing enzymes are present in other FRDA models, cerebellar tissue from YG8sR FRDA mouse models was analyzed (Figure S1). A significant decrease in *Sphk2* expression (*p* = 0.0163), along with a trending decrease in *Sphk1* expression (*p* = 0.0546), was observed at the mRNA level (Figure S1A), while a significant increase in *Lpp1* (*p* = 0.0123) and *Lpp3* mRNA (*p* = 0.0450) expression was found (Figure S1B). Protein expression explored using western blot showed increases in Lpp1 (*p* = 0.0057) and decreases in Sphk1 (*p* = 0.0075) protein expression in YG8sR mouse cerebellum tissues (Figures S1C and S1D), in line with the mRNA data. The results from YG8sR mouse cerebellum tissues are consistent with the findings from human FRDA fibroblast cell lines.

### Targeting SPHK1 and LPP1 led to an improvement in frataxin expression in human FRDA fibroblast cell lines

To boost Cer metabolism toward the synthesis of C1P and/or S1P, we used specific kinase activators or inhibitors to address how they might affect the FRDA pathogenesis. We first investigated the effect on frataxin expression levels. Human fibroblast cell lines were treated with an *SPHK1* activator, K6PC-5 (Sigma-Aldrich), or an *LPP1* inhibitor, XY-14 (Echelon Biosciences). The safety and tolerability of both compounds were tested using PrestoBlue Cell Viability Assay (Figure S2). No K6PC-5-induced toxicity was observed after 24 (Figure S2A) or 48 (Figure S2B) h. Following 72 h, 100 μM (*p* = 0.0154) and 200 μM (*p* = 0.0361) K6PC-5 were found to be toxic (Figure S2C). Similarly, toxicity was not observed after 24 (Figure S2D) or 48 (Figure S2E) h, but 10 μM XY-14 was toxic (*p* = 0.0384) following 72-h treatment (Figure S2F). Based on these results, 72-h treatment of 10 μM K6PC-5, 0.1 μM XY-14, or 1 μM XY-14 was selected for further experiments.

**Figure 2:**
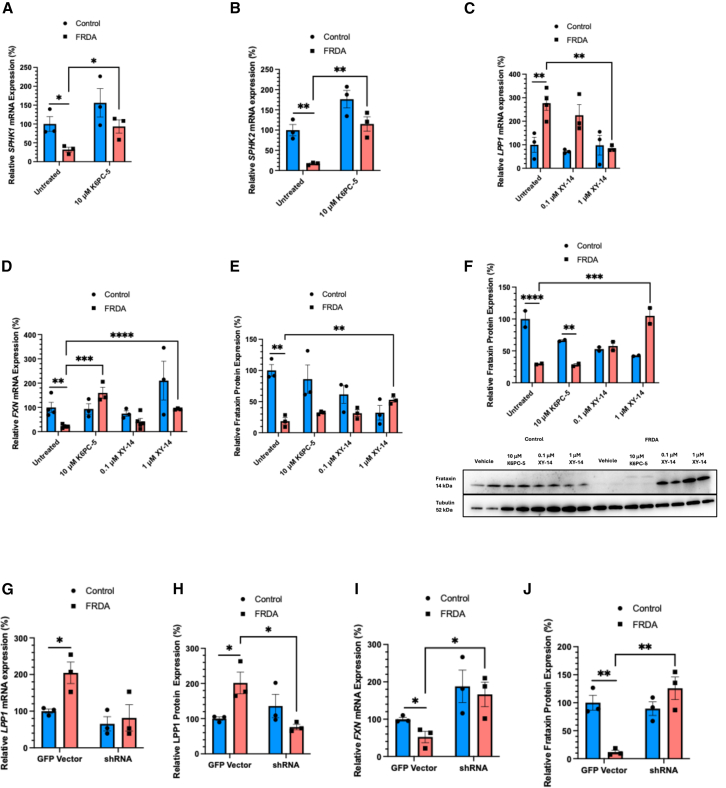
Analysis of frataxin, SPHK, and LPP expression levels following treatment with SPHK1 agonist and LPP1 inhibitor in human fibroblast cell lines (A and B) Effect of K6PC-5 on (A) *SPHK1* and (B) *SPHK2* gene expression levels, *n* = 3. (C) Effect of XY-14 on *LPP1* gene expression, *n* = 3–4. (D) Effect of K6PC-5 and XY-14 on frataxin gene expression in human fibroblast cell lines, *n* = 3–5, measured by RT-qPCR. (E) Frataxin protein expression quantified by ELISA following treatment with K6PC-5 and XY-14, *n* = 3. (F) Representative immunoblots showing the effect of K6PC-5 and XY-14 on frataxin protein expression, *n* = 2, with relative quantification of frataxin bands normalized to tubulin and control. (G and H) Effect of shRNA-mediated reduction of LPP1 on *LPP1* (G) mRNA and (H) protein expression levels and frataxin (I) mRNA and (J) protein expression levels in human fibroblast cell lines, *n* = 3, measured by RT-qPCR (mRNA) and ELISA (protein). Data are represented as mean ± SEM. Statistical significance was determined using an unpaired, two-tailed Student’s *t* test or two-way ANOVA with Tukey’s multiple comparisons test (∗*p <* 0.05, ∗∗*p <* 0.01, ∗∗∗*p <* 0.001, ∗∗∗∗*p <* 0.0001).

K6PC-5 treatment significantly increased *SPHK1* mRNA expression (Figure 2A, *p* = 0.0311) and interestingly, also significantly increased *SPHK2* mRNA expression (Figure 2B, *p* = 0.0055) in human FRDA fibroblast cell lines. A study in 2021 suggested that K6PC-5 may not be specific for *SPHK1*,^31^ and these results showed that K6PC-5 may have some effect on *SPHK2*, either directly or indirectly. A similar trend was observed with 1 μM XY-14, which significantly decreased *LPP1* mRNA expression (Figure 2C, *p* = 0.0016). Surprisingly, treatment with K6PC-5 also significantly increased the *FXN* mRNA expression in human FRDA fibroblast cell lines (*p* = 0.0002), with 1 μM XY-14 treatment simultaneously increasing *FXN* mRNA expression (*p < 0.0001*) (Figure 2D). However, changes in mRNA expression do not necessarily translate to changes in protein levels. For instance, HMTase inhibitors have been shown to increase *FXN* mRNA expression without affecting frataxin protein levels.^32^ Therefore, to determine whether K6PC-5 and XY-14 could increase frataxin protein expression, a Human Frataxin ELISA Kit was used. Results showed a significant increase in frataxin protein expression following treatment with 1 μM XY-14 (Figure 2E, *p* = 0.0075), which was corroborated by western blot analysis (Figure 2F, *p* = 0.0003). Quantification confirmed that treatment with 1 μM XY-14 significantly increased frataxin protein expression in human FRDA fibroblast cell lines. These results suggest that sphingolipids may modulate pathways controlling frataxin expression.

It has been suggested that XY-14 may impact not only LPP1 but also LPP2 and LPP3.^33^ This raised concerns as LPPs interact within various pathways, and targeting all LPPs could be detrimental. To ensure specific targeting of *LPP1*, shRNA was used to selectively decrease *LPP1* expression. Human FRDA and control fibroblast cell lines were transduced with empty vector or shRNA (LPP1 6181) lentiviruses. Reduction of *LPP1* expression following treatment was confirmed using quantitative reverse-transcription PCR (RT-qPCR) (Figure 2G, *p* = 0.0571) and ELISA (Figure 2H, *p* = 0.0171). Consistent with the results obtained from XY-14 experiments, *FXN* mRNA expression was significantly increased after shRNA-mediated reduction of LPP1 (Figure 2I, *p* = 0.0350), while frataxin protein expression also showed a significant increase (Figure 2J, *p* = 0.0051). These results suggest that *LPP1* plays a role in regulating *FXN* expression.

### Targeting SPHK1 and LPP1 led to improvements in mitochondrial function in human FRDA fibroblast cell lines

**Figure 3:**
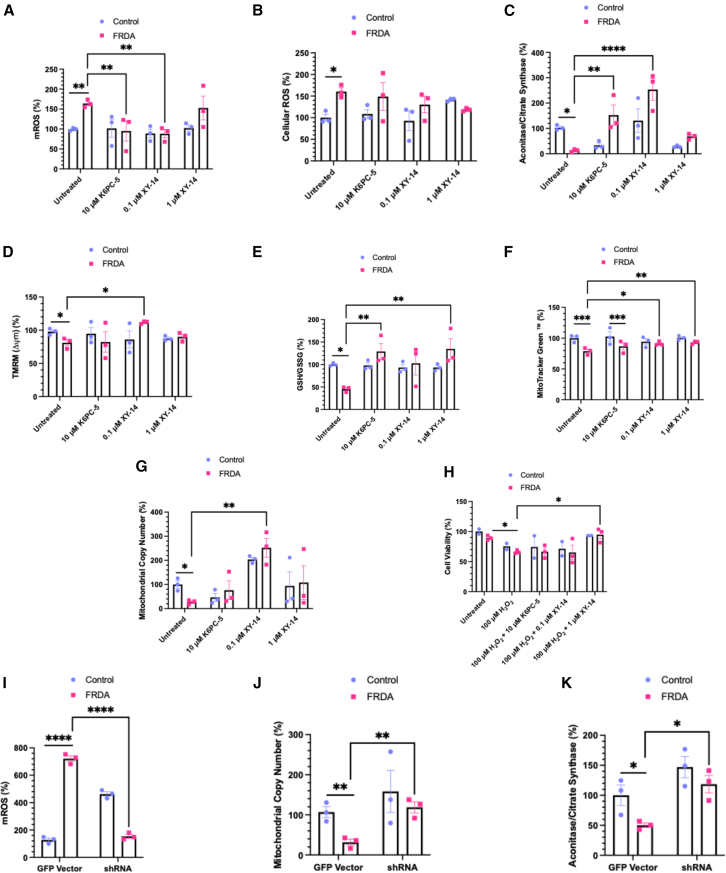
Effect of targeting *SPHK1* and *LPP1* on mitochondrial dysregulation in human FRDA fibroblast cell lines (A–H) Effect of K6PC-5 and XY-14 on (A) mitochondrial ROS (mROS) quantified using MitoSOX Red, *n* = 3; (B) total cellular ROS measured using CM-H_2_DCFDA, *n* = 3; (C) aconitase activity measured usnig the Aconitase Assay Kit and normalized to citrate synthase activity, *n* = 3; (D) mitochondrial membrane potential (ΔΨm), measured using TMRM, *n* = 3; (E) GSH/GSSG ratio measured using the Glutathione Fluorescent Detection Kit, *n* = 3; (F) mitochondrial mass, measured using MitoTracker Green FM Dye, *n* = 3; (G) mitochondrial DNA (mtDNA) copy number, quantified using RT-qPCR, *n* = 3; and (H) H_2_O_2_-induced oxidative stress, assessed using PrestoBlue Cell Viability Reagent, *n* = 2–3. (I–K) Effect of shRNA-mediated reduction of LPP on (I) mROS, measured using MitoSOX Red, *n* = 3; (J) mtDNA copy number, quantified using RT-qPCR, *n* = 3; and (K) aconitase activity measured usnig the Aconitase Assay Kit, normalized to citrate synthase activity in human fibroblast cell lines, *n* = 3. Data are represented as mean ± SEM. Statistical significance was determined using an unpaired, two-tailed Student’s *t* test or two-way ANOVA with Tukey’s multiple comparisons test or Holm-Šídák’s multiple comparisons test (∗*p <* 0.05, ∗∗*p <* 0.01, ∗∗∗*p <* 0.001, ∗∗∗∗*p <* 0.0001).

To investigate the relationship between sphingolipid metabolism and mitochondrial dysfunction in FRDA, human fibroblast cell lines were treated with K6PC-5, XY-14, or shRNA to reduce *LPP1* expression. Oxidative stress is a well-known hallmark of FRDA.^7^ The effects of K6PC-5 and XY-14 on ROS levels were assessed. After 72 h of treatment, cells were stained with either MitoSOX Red to measure mitochondrial ROS (mROS) or CM-H_2_DCFDA for total ROS (tROS). Notably, treatment with 10 μM K6PC-5 (*p* = 0.0040) and 0.1 μM XY-14 (*p* = 0.0024) resulted in a decrease in mROS levels (Figure 3A), while no significant effect was observed on tROS levels (Figure 3B). Aconitase activity, which is known to be reduced in FRDA,^34^ was significantly increased following treatment with K6PC-5 (*p* = 0.0097) and XY-14 (*p* < 0.0001) in human FRDA fibroblast cell lines (Figure 3C). In addition, treatment with 0.1 μM XY-14 improved mitochondrial membrane potential (ΔΨm) (Figure 3D, *p* = 0.0329).

Since glutathione (GSH) is protective against ROS,^16^ the GSH/GSSG ratio was measured in both control and FRDA fibroblast cell lines. Treatment with both K6PC-5 (*p* = 0.0038) and XY-14 (*p* = 0.0021) resulted in a significant improvement in the glutathione level (Figure 3E). Treatment with 0.1 μM (*p* = 0.0106) and 1 μM XY-14 (*p* = 0.0057) led to significant improvements in mitochondrial mass (Figure 3F) and 0.1 μM XY-14 led to significant improvements in mitochondrial DNA (mtDNA) copy number (Figure 3G, *p* = 0.0040). To assess whether the compounds could prevent cell death induced by oxidative stress, the cells were incubated with 100 μM H_2_O_2_ for 1 h following treatment, a condition that significantly reduces viability in human FRDA cell lines (*p* = 0.0418). Cell viability was assessed 24 h later, and it was found that 1 μM XY-14 significantly increased cell survival (Figure 3H, *p* = 0.0299). To investigate whether shRNA-mediated reduction of *LPP1* affects mitochondrial dysfunction, mROS, mtDNA copy number, and aconitase activity were measured in control and FRDA fibroblast cell lines transduced with either empty vector or shRNA. Interestingly, a significant decrease in mROS (*p* < 0.0001) was found in FRDA fibroblasts following shRNA-mediated reduction of *LPP1* (Figure 3I). Furthermore, a significant increase in both mtDNA copy number (Figure 3J, *p* = 0.0055) and aconitase activity (Figure 3K, *p* = 0.0105) was found in FRDA fibroblast cell lines treated with shRNA. Overall, these results suggest that sphingolipids play a role in mitochondrial function, and targeting sphingolipid-metabolizing enzymes could positively influence mitochondrial processes affected in FRDA.

### Targeting SPHK1 and LPP1 led to an improvement in *Nrf2* expression and labile iron in human FRDA fibroblast cell lines

**Figure 4:**
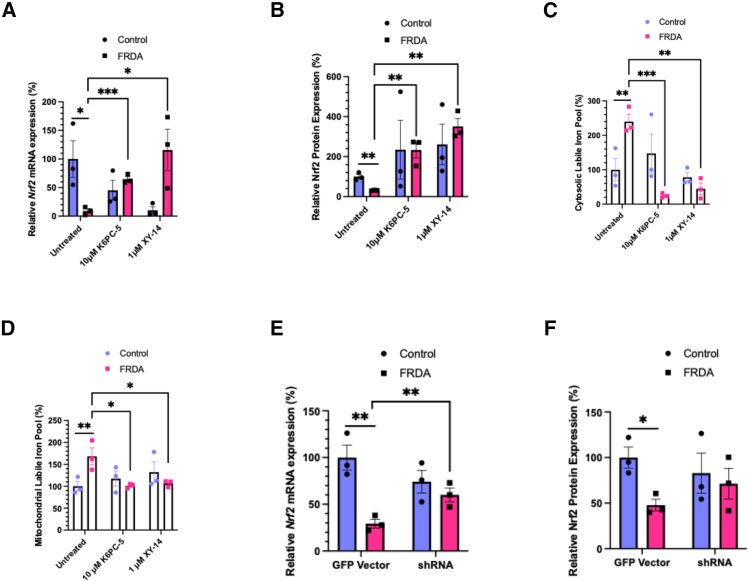
Effect of treatment with SPHK agonist and LPP inhibitor on Nrf2 expression and LIP levels in human FRDA fibroblast cell lines (A–D) Effect of K6PC-5 and XY-14 on (A) *Nrf2* gene expression, measured using RT-qPCR; (B) Nrf2 protein expression, quantified using ELISA; (C) cLIP measured using Cytosense LI (CY6); and (D) mLIP measured using Mitosense LI (M) in human fibroblast cell lines, *n* = 3. (E and F) Effect of shRNA-mediated reduction of LPP on Nrf2 (E) gene expression measured using RT-qPCR and (F) protein expression quantified by ELISA, in human fibroblast cell lines *n* = 3. Data are represented as mean ± SEM. Statistical significance was determined using an unpaired, two-tailed Student’s *t* test or two-way ANOVA with Tukey’s multiple comparisons test (∗*p <* 0.05, ∗∗*p <* 0.01, ∗∗∗*p <* 0.001).

Given that Nrf2 expression is dysregulated in FRDA and is the target of the newly approved drug, omaveloxelone, the effects of K6PC-5 and XY-14 on *Nrf2* mRNA expression were explored. Since 10 μM K6PC-5 and 1 μM XY-14 successfully increased *FXN* expression, these concentrations were taken forward for investigation on *Nrf2* expression. Interestingly, both treatment with K6PC-5 (*p* = 0.0008) and XY-14 (*p* = 0.0429) led to a significant increase in *Nrf2* mRNA levels (Figure 4A). K6PC-5 (*p* = 0.0070) and XY-14 (*p* = 0.0012) also exerted this effect at the protein level (Figure 4B) in human FRDA fibroblast cell lines. These findings align with other studies that demonstrated a relationship between sphingolipids and Nrf2 expression,^27^^,^^28^ further supporting the potential of targeting sphingolipids to mitigate oxidative stress.

Closely linked to oxidative stress, another hallmark of FRDA is the dysregulation of iron metabolism, primarily characterized by mitochondrial iron accumulation and reduced activity of iron-sulfur cluster enzymes.^35^^,^^36^ To investigate whether K6PC-5 and XY-14 affect iron levels, CY6 was used for measurement of the cytosolic labile iron pool (cLIP) and Mitosense was used for measurement of the mitochondrial labile iron pool (mLIP). Cytosolic iron has been somewhat explored in FRDA, although conclusions regarding alterations remain inconclusive.^37^ In our study, FRDA fibroblast cell lines exhibited significantly higher iron levels compared to controls, and treatment with 10 μM K6PC-5 (*p* = 0.0007) and 1 μM XY-14 (*p* = 0.0015) significantly reduced cLIP (Figure 4C). These concentrations of K6PC-5 (*p* = 0.0210) and XY-14 (*p* = 0.0327) also significantly reduced mLIP (Figure 4D). These findings suggest that sphingolipids may influence iron regulation and that targeting them could reduce oxidative stress by modulating iron levels.

Moreover, *Nrf2* expression was explored in both human control and FRDA fibroblast cell lines following shRNA treatment. A significant increase in *Nrf2* mRNA expression (*p* = 0.0248) was observed in shRNA-treated human FRDA fibroblast cell lines (Figure 4E); however, this increase was not reflected in Nrf2 protein expression (Figure 4F). This discrepancy suggests that other enzymes or pathways may also contribute to Nrf2 expression beyond LPP1.

### Altered SPHK and LPP expression in other FRDA *in vitro* models

Although human FRDA fibroblast cell lines derived from patient skin are widely used as an *in vitro* model for studying FRDA, they have the limitation of not representing the cell types most commonly affected by the disease, such as neurons and cardiomyocytes. Therefore, to assess alterations in SPHK and LPP, as well as the effect of targeting these enzymes, we utilized peripheral sensory neurons (PSNs) and cardiomyocytes (CMs) differentiated from induced pluripotent stem cells (iPSCs) of FRDA patients and relative isogenic control.

Human FRDA iPSCs and their relative isogenic controls were differentiated into PSNs. Successful differentiation to iPSC-derived PSNs was determined using immunofluorescence to confirm the expression of Peripherin and Brn3a (Figure S3). RT-qPCR was also used to confirm the pluripotency of iPSCs (Figures S4A and S4B) and subsequent differentiation to PSNs (Figures S4C and S4D). Following this, the cell tolerability and safety of K6PC-5 was tested using PrestoBlue Cell Viability Assay (Figure S4E), and 10 μM K6PC-5 was taken forward. This method was also used to evaluate the tolerability and safety of XY-14 (Figure S4F), and 1 μM XY-14 was taken forward.

**Figure 5:**
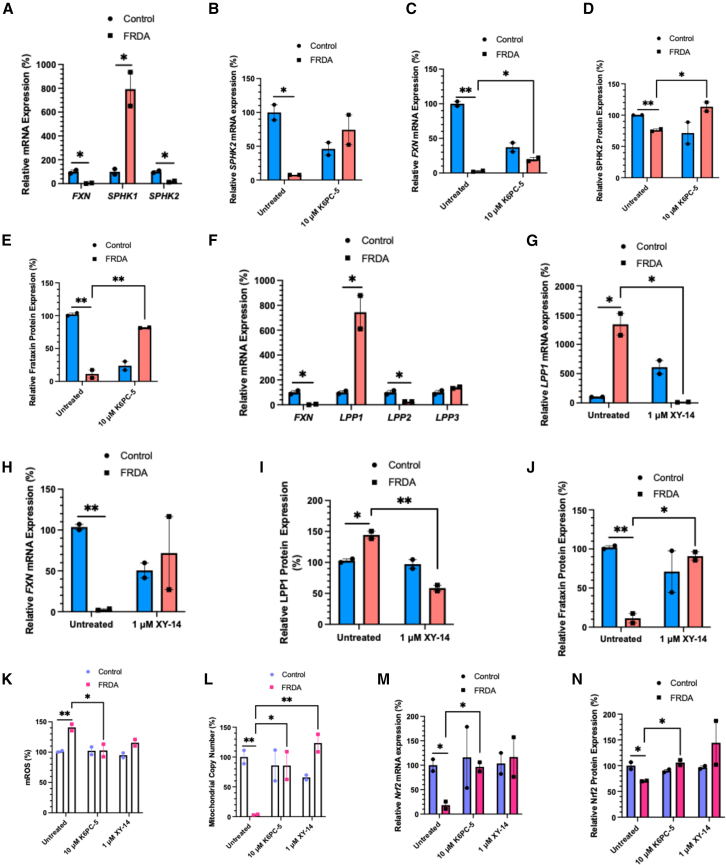
Sphingolipid dysregulation and the effect of K6PC-5 and XY-14 on FXN expression in FRDA iPSC-derived PSNs (A–C) RT-qPCR analysis of (A) SPHK1/2 gene expression, *n* = 2; (B) effect of K6PC-5 on SPHK2 gene expression, *n* = 2; and (C) FXN gene expression, *n* = 2. (D and E) ELISA quantification of (D) SPHK2 protein expression, *n* = 2, and (E) frataxin protein expression, *n* = 2. (F–H) RT-qPCR analysis of (F) LPP1/2/3 gene expression, *n* = 2; (G) effect of XY-14 on LPP1 gene expression, *n* = 2; and (H) FXN gene expression, *n* = 2. (I and J) ELISA quantification of (I) LPP1 protein expression, *n* = 2, and (J) frataxin protein expression, *n* = 2. (K–N) Effect of K6PC-5 and XY-14 on (K) mROS, measured using MitoSOX Red, *n* = 2; (L) mtDNA copy number, measured using RT-qPCR, *n* = 2; (M) Nrf2 gene expression, measured using RT-qPCR, *n* = 2; and (N) Nrf2 protein expression, quantified by ELISA, *n* = 2. Data are represented as mean ± SEM. Statistical significance was determined using an unpaired, two-tailed Student’s *t* test or two-way ANOVA with Tukey’s multiple comparisons test (∗*p* < 0.05, ∗∗*p* < 0.01).

To identify if *SPHK* is also dysregulated in human FRDA PSNs, the mRNA expression (Figure 5A) was quantified using RT-qPCR. A significant increase in *SPHK1* (*p* = 0.0404) and a significant decrease in *SPHK2* (*p* =0.0155) mRNA expression was observed. Treatment with K6PC-5 led to a trend of increased *SPHK2* mRNA expression (Figure 5B, *p* = 0.0948) and significantly increased expression of *FXN* (Figure 5C, *p* = 0.0245). K6PC-5 also significantly increased SPHK2 (Figure 5D, *p* = 0.0368) and frataxin (Figure 5E, *p* = 0.0076) protein expression. The same investigation was carried out for *LPP*. *LPP1* mRNA expression was shown to be increased (*p* = 0.0410), while *LPP2* mRNA expression was decreased (*p* = 0.0345) (Figure 5F). XY-14 decreased the mRNA expression of *LPP1* (Figure 5G, *p* = 0.0191) but did not have any significant effect on *FXN* mRNA expression (Figure 5H). However, treatment with XY-14 in the FRDA PSNs led to a significant reduction in LPP1 protein expression (Figure 5I, *p* = 0.0077) and a significant increase in frataxin protein expression (Figure 5J, *p* = 0.0103). Overall, these results indicate that *SPHK* and *LPP* could be involved in the regulation of frataxin expression.

To determine whether K6PC-5 and XY-14 affect mitochondrial processes and antioxidant responses, their impact on mROS, mtDNA copy number, and Nrf2 expression was assessed in FRDA PSNs. K6PC-5 significantly improved mROS levels (Figure 5K, *p* = 0.0104), and both K6PC-5 (*p* = 0.0264) and XY-14 (*p* = 0.0046) significantly increased mtDNA copy number (Figure 5L). Interestingly, K6PC-5 significantly increased both *Nrf2* mRNA (Figure 5M, *p* = 0.0222) and protein (Figure 5N, *p* = 0.0184) expression levels. Given that FRDA PSNs represent a more relevant cell type for FRDA research, these findings suggest that targeting *SPHK* and *LPP* could be an effective strategy for mitigating the mitochondrial dysfunction that is observed in FRDA patients.

To investigate *SPHK* and *LPP* in a cell type relevant to the disease, human FRDA iPSCs and their relative isogenic controls were also differentiated into CMs. Following confirmation of differentiation (Figures S5A–S5D), the *SPHK2* and *LPP1* mRNA expression levels were evaluated. A non-significant decrease in *SPHK1* mRNA expression (*p* = 0.1896) was observed (Figure S5E), while *LPP2* expression was significantly decreased (*p* = 0.0282) and *LPP3* expression was significantly increased (*p = 0.0481*) (Figure S5F) in FRDA iPSC-derived CMs compared to control.

### Targeting Sphk1 and Lpp1 *in vivo*

Having determined that *Sphk* and *Lpp* are altered both *in vitro* and in YG8sR mouse cerebellum tissue, a preliminary *in vivo* study was carried out to assess the safety and efficacy of K6PC-5 and XY-14 in YG8sR mice. K6PC-5 has previously been administered via intraperitoneal (i.p.) injection in the R6/2 Huntington’s disease mouse model and was shown to be safe at a dose of 0.05 mg/kg/d.^38^^,^^39^ To our knowledge, XY-14 has not previously been administered in any mouse model by any route; therefore, this study represents the first assessment of its safety when administered at 0.05 mg/kg/d and 0.1 mg/kg/d over a 4-day period in the YG8sR mouse model. The effects of K6PC-5 and XY-14 were investigated in cerebellum tissue from YG8sR mice following daily i.p. administration for 4 days, with Y47R mice serving as controls.

**Figure 6:**
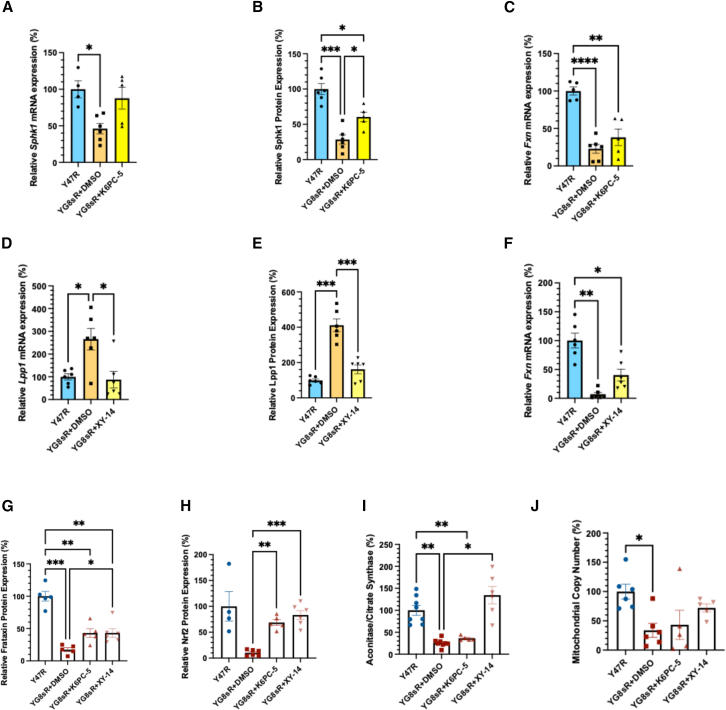
Sphingolipid dysregulation and the effect of K6PC-5 and XY-14 on FXN expression in YG8sR mouse cerebellum tissue (A) Effect of K6PC-5 on Sphk1 mRNA expression, measured using qRT-PCR, *n* = 4–6. (B and C) (B) Sphk1 protein expression, quantified by ELISA, *n* = 5–6, and (C) Fxn mRNA expression, measured using RT-qPCR, *n* = 5–6. (D–F) Effect of XY-14 on (D) Lpp1 mRNA expression, measured using RT-qPCR, *n* = 6; (E) Lpp1 protein expression, quantified by ELISA, *n* = 6; and (F) Fxn mRNA expression, determined using RT-qPCR, *n* = 6. (G) Frataxin protein expression following treatment with K6PC-5 and XY-14, quantified by ELISA, *n* = 5–6. (H) Nrf2 protein expression following treatment with K6PC-5 and XY-14, quantified using ELISA, *n* = 5–6. (I) Aconitase activity measured using the Aconitase Assay Kit and normalized to citrate synthase activity, *n* = 5–6, and (J) mtDNA copy number, measured using RT-qPCR, *n* = 5, following K6PC-5 or XY-14 treatment. Statistical significance was determined using Welch’s *t* test or Welch’s ANOVA with Dunnett’s T3 multiple comparisons test (∗*p* < 0.05, ∗∗*p* < 0.01, ∗∗∗*p* < 0.001, ∗∗∗∗*p* < 0.0001).

To assess the effect of K6PC-5 in YG8sR mouse cerebellum tissue, RT-qPCR was performed. *Sphk1* mRNA expression was significantly reduced in YG8sR mouse cerebellum tissue compared to Y47R controls (Figure 6A, *p* = 0.0283), and expression returned to endogenous levels following treatment with 0.05 mg/kg/d K6PC-5. However, K6PC-5 treatment did not improve *Fxn* mRNA expression, which remained significantly reduced following treatment (Figure 6C, *p* = 0.0064).

Sphk1 protein expression following treatment with 0.05 mg/kg/d K6PC-5 was quantified using ELISA (Figure 6B). Sphk1 protein levels were significantly increased in YG8sR mouse cerebellum tissue following treatment (*p* = 0.0226) but remained significantly lower than endogenous levels observed in Y47R mice (*p* = 0.0117).

The same experimental approach was used to investigate the effect of XY-14 in YG8sR mouse cerebellum tissue. RT-qPCR analysis demonstrated that *Lpp1* mRNA expression was significantly decreased to the level found in endogenous control following 0.1 mg/kg/d of XY-14 (Figure 6D, *p* = 0.0425). A trend toward increased *Fxn* mRNA expression was observed following XY-14 treatment (Figure 6F, *p* = 0.0570).

Lpp1 protein expression following treatment with 0.1 mg/kg/d XY-14 was quantified by ELISA (Figure 6E). Lpp1 protein levels were significantly elevated in YG8sR mouse cerebellum tissue compared to Y47R (*p* = 0.0004) and returned to endogenous levels following treatment (*p* = 0.0008).

Frataxin protein expression was also assessed using ELISA (Figure 6G). As previously established, frataxin protein levels were reduced in YG8sR mouse cerebellum tissue compared to Y47R controls (*p* = 0.0002). Treatment with both K6PC-5 and XY-14 improved frataxin expression in YG8sR mice, with the increase following XY-14 treatment reaching statistical significance (*p* = 0.0478) compared to DMSO-treated YG8sR controls. However, frataxin levels did not reach the endogenous levels observed in Y47R control mice.

In addition, the effects of K6PC-5 and XY-14 on mitochondrial function *in vivo* were investigated by assessing Nrf2 levels (Figure 6H), aconitase activity (Figure 6I), and mtDNA copy number (Figure 6J). Nrf2 levels were significantly increased following treatment with both compounds (K6PC-5, *p* = 0.0011; XY-14, *p* = 0.0009). Aconitase activity was significantly increased following XY-14 treatment (*p* = 0.0129); however, no significant changes in mtDNA copy number were observed following treatment with either K6PC-5 or XY-14. Overall, these findings suggest a potential effect on frataxin expression and mitochondrial function by targeting sphingolipid synthesis; however, additional long-term studies are required to confirm these effects.

## Discussion

Altered sphingolipid levels, particularly elevated Cers, have been implicated in several neurodegenerative diseases, including FRDA. Pioneering work by Chen et al. demonstrated increased sphingolipid levels in the hearts of FRDA patients, with further evidence of altered sphingolipid metabolism in *Drosophila* and mouse models following frataxin loss.^10^^,^^11^ Similarly, Wang et al. reported sphingolipid dysregulation in human FRDA fibroblasts.^30^ In this study, we present evidence suggesting that the dysregulation of sphingolipid-metabolizing enzymes, particularly SPHK and LPP, may contribute to FRDA progression by affecting key cellular processes, including mitochondrial function, iron metabolism, and oxidative stress resistance. Moreover, targeting these enzymes appears to modulate several hallmarks of FRDA, offering potential new therapeutic avenues for managing FRDA symptoms.

The data obtained from untreated human FRDA and control fibroblast cell lines indicate that there may be changes in sphingolipid-metabolizing enzymes, particularly changes in the expression of *SPHK* and *LPP*. Changes in the sphingolipids themselves have been shown in human FRDA fibroblasts already,^30^ as well as in *Drosophila*.^10^ However, by focusing on the expression of the enzymes that regulate these sphingolipids, our results suggest that these enzymes play a central role in the observed sphingolipid dysregulation.

Our analysis of the expression of sphingolipid-metabolizing enzymes revealed that SPHK and LPP are the key enzymes with altered levels in FRDA fibroblast cell lines. These enzymes play a crucial role in the sphingolipid rheostat, as they catalyze the interconversion of sphingolipids that either activate or inhibit pathways governing cell survival or apoptosis. Specifically, FRDA cells exhibit downregulation of SPHK and upregulation of LPP. A reduction in SPHK activity could result in lower S1P levels, which may, in turn, diminish the activation of pathways such as the PI3K-Akt pathway, crucial for promoting cell proliferation and survival.^21^ Furthermore, increased LPP activity suggests elevated Cer levels, as LPP dephosphorylates C1P to Cer, potentially enhancing the activation of ROS-generating pathways.^26^ Since LPP also targets S1P and LPA,^25^ its upregulation could have broader effects, ultimately hindering cell survival and proliferation.

Treatment with K6PC-5 increased *FXN* mRNA expression, while XY-14 treatment led to significant increases in both *FXN* mRNA and protein levels in human FRDA fibroblast cell lines. This is particularly interesting as it suggests a potential link between sphingolipid metabolism and frataxin expression, highlighting sphingolipid pathways as promising therapeutic targets for FRDA. We propose that the observed increase in *FXN* expression following targeting of sphingolipid metabolism may, at least in part, be mediated through effects on *Nrf2* signaling. Within this framework, sphingolipid-driven alterations in lipid signaling and cellular redox balance may enhance Nrf2 activity and its downstream transcriptional programs, thereby creating a permissive environment for cytoprotective gene expression. Mechanistically, AREs, the *cis*-regulatory sequences bound by *Nrf2*, have been identified within the *FXN* locus, including multiple sites upstream of the transcription start site (TSS).^39^ This raises the possibility that enhanced Nrf2 activity could facilitate increased binding to AREs within or proximal to the *FXN* locus, thereby promoting *FXN* transcription. Consistent with this, Nrf2 activation has been associated with improved mitochondrial function and increased aconitase activity in studies using compounds such as dyclonine.^39^^,^^40^

Importantly, the extent to which Nrf2 activation drives *FXN* expression appears to depend on the broader cellular context. For example, dimethyl fumarate (DMF), a clinically approved Nrf2 activator, has been shown to significantly increase transcription initiation at the *FXN* locus in FRDA patient-derived cells,^20^^,^^41^ an effect associated with reduced R-loop formation and transcriptional pausing.^41^ Moreover, in a clinical setting, DMF-treated multiple sclerosis patients exhibited an approximate 85% increase in *FXN* expression,^41^ supporting the translational relevance of pharmacological *FXN* modulation in humans.

Similarly, modulation of cellular redox status using antioxidant compounds such as L-ascorbic acid and N-acetylcysteine has also been reported to increase FXN mRNA and protein levels in FRDA models,^3^^,^^20^ further supporting a role for redox-dependent mechanisms in *FXN* regulation.

In contrast, more selective NRF2 activators such as omaveloxolone have generally been associated with relatively modest increases in frataxin expression.^42^ This distinction suggests that Nrf2 activation alone may be insufficient to drive robust *FXN* upregulation and supports the possibility that sphingolipid modulation may act upstream of, or in parallel with, Nrf2 signaling, engaging additional regulatory mechanisms that collectively enhance *FXN* expression.

In our investigation, targeting *SPHK* and *LPP* in human FRDA fibroblast cell lines led to significant improvements in the mitochondrial dysregulation that is associated with the disease. Considering that mitochondrial dysfunction and subsequent oxidative stress are hallmark features of FRDA, we focus on key markers of mitochondrial functionality, including aconitase activity, mitochondrial membrane potential (ΔΨm), mitochondrial mass, mtDNA copy number, and redox status, specifically assessing mROS, tROS, and the GSH/GSSG ratio. Remarkably, treatment with XY-14 resulted in significant enhancements across all parameters, except for tROS levels. shRNA-mediated targeting of *LPP1* led to similar results. In contrast, K6PC-5 notably reduced mROS, increased aconitase activity, and improved the GSH/GSSG ratio. These findings underscore the crucial role of sphingolipids in maintaining mitochondrial function in FRDA.

Previous studies have shown that Cer and Sph are involved in promoting apoptotic processes within cells.^43^ In the presence of tumor necrosis factor alpha (TNF-α), Cers accumulate within mitochondrial membranes, allowing permeabilization of the outer mitochondrial membrane and the release of apoptotic mediators.^43^ In addition, Cers contribute to apoptosis by inactivating ERK that increases ROS levels, including nitric oxide synthase, NADPH oxidase, and xanthine oxidase.^26^ In particular, the interaction between Cer and NADPH oxidase has been studied in macrophages, endothelial cells, and smooth muscle cells of the aorta, revealing that Cer enhances the production of superoxide (O_2_^−^).^26^

Treatment with K6PC-5 and XY-14 led to improved Nrf2 gene and protein expression. The effect of K6PC-5 may be attributed to the interaction between *SPHK1* and *Nrf2*, which has been shown to be activated by K6PC-5 in SH-SY5Y cells.^28^ In that study, SH-SY5Y cells were subjected to oxygen-glucose deprivation and reoxygenation, leading to an increase in *SPHK1* expression, while K6PC-5 was found to increase *SPHK1* activity.^28^ K6PC-5 also increased the expression of Nrf2-dependent genes, including the glutamylcysteine synthetase catalytic subunit (GCLC), NADPH quinone oxidoreductase 1 (NQO1), and heme oxygenase 1 (HO1), all of which are involved in promoting antioxidant mechanisms. In addition, it upregulated Nrf2 protein expression itself.^28^ Therefore, it can be inferred that K6PC-5 activates Nrf2, which in turn improves aconitase activity and GSH/GSSG balance, although it may not have a significant impact on other mitochondrial processes. The effects exerted by XY-14 could be related to the sphingolipids targeted by LPP. LPP regulates the expression of several sphingolipids, but its ability to reduce C1P and LPA levels specifically may underlie XY-14’s effect on FXN and Nrf2 expression. It is known that LPA can stabilize Nrf2 expression,^25^ and more recently, it was shown that C1P inhibits the interaction between Keap1 and Nrf2.^44^ This study, focused on acute liver injury, demonstrated that both C1P and Keap1 bind to the double glycine repeat (DGR) domain within Nrf2. C1P binding allows Nrf2 to dissociate from Keap1, facilitating its nuclear translocation and the initiation of antioxidant mechanisms.^44^ Therefore, it is possible that XY-14’s inhibition of LPP1 leads to higher LPA and C1P levels, stabilizing Nrf2 expression and enabling the C1P-Nrf2 interaction, which promotes antioxidant mechanisms, improves mitochondrial function, and increases FXN expression.

These results identify that sphingolipid-metabolizing enzymes do play a role in oxidative stress and *FXN* expression. However, it is unknown if the effects of K6PC-5 and XY-14 are exhibited more upstream or downstream of *FXN* expression and oxidative stress. The relationship between mitochondrial homeostasis and frataxin expression in FRDA is inherently complex and cannot be readily explained by a single unidirectional mechanism. Our findings support a bidirectional and partially parallel model. In this model, dysregulation of sphingolipid metabolism represents an upstream pathological driver capable of independently influencing both mitochondrial integrity and *FXN* expression.^10^^,^^26^ In the disease state, alterations in SPHK and LPP activity are expected to impair mitochondrial homeostasis by shifting Cer-, C1P-, and S1P-dependent signaling pathways that regulate redox balance, iron toxicity, and apoptotic susceptibility.^26^ These effects occur in the context of reduced frataxin levels and contribute to mitochondrial vulnerability in FRDA. However, under pharmacological or experimental modulation of these enzymes, sphingolipid-driven stress signaling can have a different consequence. It may create a transcriptionally permissive environment that facilitates increased *FXN* expression, for example, via NRF2-dependent mechanisms.^3^^,^^27^^,^^39^^,^^40^^,^^41^^,^^42^^,^^43^^,^^44^ Elevated frataxin levels could then further reinforce mitochondrial function by improving iron-sulfur cluster biogenesis and limiting oxidative stress.^39^^,^^40^ These observations suggest that sphingolipid dysregulation participates in a feedforward regulatory loop, in which pathological changes impair mitochondrial function, while targeted modulation of the same pathways can activate compensatory transcriptional programs that restore *FXN* expression, rather than a single unidirectional causal pathway.

The study carried out in *fh*-mutant *Drosophila* concluded that iron toxicity triggers sphingolipid synthesis and contributes to neurodegeneration, and this was shown through addition of myriocin to these *Drosophila* models in iron-rich and iron-low diets.^10^ Myriocin has also been investigated by other researchers in the fields of Alzheimer’s disease and Parkinson’s disease, and a study published in 2022 identified that hypoxia-related transcription factors were most likely to be activated by myriocin and that the HIF-1 signaling pathway is the most likely target of myriocin to exerts its cytoprotective effects.^45^ This could be the pathway by which other drugs that target sphingolipid-metabolizing enzymes, such as K6PC-5 and XY-14, reduce iron toxicity.

**Figure 7:**
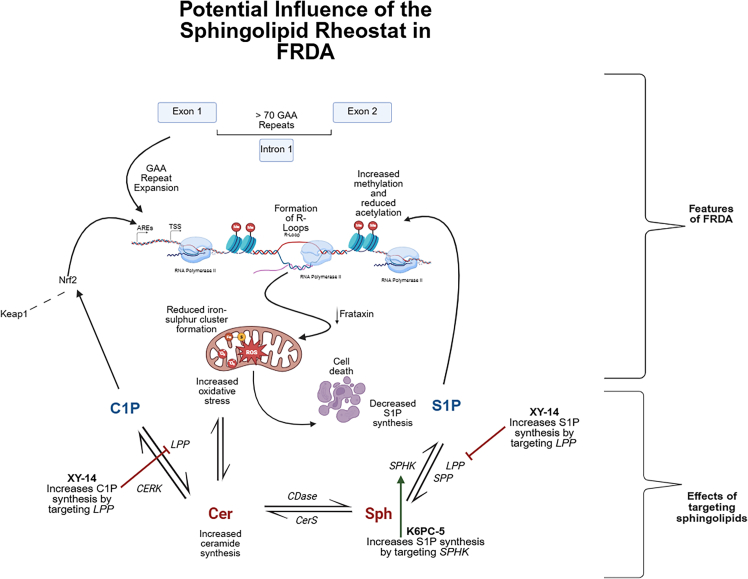
Proposed mechanism of the effects of K6PC-5 and XY-14 in FRDA models The GAA repeat expansion within the FXN gene leads to the formation of R-loops, increased DNA methylation, and impaired Fe-S cluster formation, resulting in hallmark features of FRDA such as mitochondrial dysfunction, oxidative stress, and cell death. Altered sphingolipid levels have been reported in several publications.^10^^,^^11^^,^^30^ We propose that this altered sphingolipid profile arises from dysregulation of sphingolipid-metabolizing enzymes, particularly SPHK and LPP. Targeting these enzymes leads to greater S1P and C1P formation. Increasing SPHK expression boosts S1P formation via SPHK2, which promotes histone acetylation and increases FXN expression. Simultaneously, reducing LPP expression limits sphingosine production, allowing for greater S1P accumulation. In addition, SPHK activation has been linked to increased Nrf2 expression,^28^ suggesting that K6PC-5 might trigger Nrf2-mediated antioxidant defences. Furthermore, C1P has been shown to interact with Nrf2, promoting dissociation from Keap1 and promoting nuclear translocation.^44^ Targeting SPHK and LPP could thus enhance Nrf2-dependent antioxidant pathways while also binding to AREs upstream of the FXN promoter, further inducing FXN expression. Finally, modulating these enzymes may mitigate iron toxicity, potentially reducing oxidative stress in FRDA. Sphingolipids associated with apoptosis (Cer and Sph) are shown in red, and sphingolipids associated with proliferation (C1P and S1P) are shown in blue. Schematic created using BioRender.

To further elucidate the specific role of *LPP1*, especially in light of evidence from 2003 suggesting that XY-14 may also target *LPP2* and *LPP3,*^33^ and given its superior efficacy relative to K6PC-5, shRNA-mediated reduction of *LPP1* was examined in human FRDA and control fibroblast cell lines. In mouse models, deficiency of the Lpp3-coding gene, *Plpp3*, results in embryonic lethality,^46^ highlighting the importance of ensuring that experimental interventions specifically target LPP1. Consistent with this requirement, shRNA-mediated reduction of LPP1 produced effects similar to those observed with XY-14 treatment. In human FRDA fibroblast, Lpp1 knockdown led to increased *FXN* mRNA and protein expression, elevated *Nrf2* mRNA levels, reduced mROS, and improvements in mtDNA copy number and aconitase activity. These findings strengthen the conclusion that reducing *LPP1* expression enhances mitochondrial function and *FXN* expression, likely through modulation of C1P and LPA levels. The proposed mechanism is summarized in Figure 7.

However, while fibroblasts are a popular choice for cell models in FRDA research due to its ease of culture, low maintenance costs, and their characteristic increase in GAA repeats alongside reduced *FXN* expression,^47^ they are not the most representative cell type for this neurodegenerative disorder. Therefore, we extended our study to include human FRDA and control iPSC-derived PSNs. The FRDA and control iPSCs used were isogenic pairs, ensuring that any observed differences were attributable specifically to the GAA repeat expansion and consequent *FXN* expression changes. In the control lines, the GAA repeats had been excised from their corresponding FRDA counterparts, with the resulting increase in *FXN* expression previously confirmed.^48^ Prior to experimentation with K6PC-5 and XY-14, we characterized the PSNs for PRPH and Brn3a expression, confirming that these neurons exhibited significantly higher levels of both proteins compared to the iPSCs. The mRNA expression of *SPHK* and *LPP* was measured and showed increased *SPHK1* and *LPP1* mRNA expression, as well as decreased *SPHK2* and *LPP2* mRNA expression. *SPHK1* expression was only elevated in PSNs. In mouse studies, *SPHK1* has been shown to be highly expressed in leukocytes, spleen, and lung, while *SPHK2* shows higher expression in the kidneys and liver,^49^^,^^50^ indicating that *SPHK* expression across tissues is variable and perhaps not ordinarily highly expressed in tissue types widely affected by FRDA. It is more likely that these enzymes may normally be expressed at a lower level, consistent with previous reports identifying both *SPHK1* and *SPHK2* in murine DRG.^51^^,^^52^ Recent investigations have shown that loss of the peroxisomal enzyme acyl-CoA oxidase 1 (ACOX1) in glia of the CNS leads to an increase in very-long chain fatty acids (VLCFAs) in *Drosophila*.^53^^,^^54^ Increased VLCFA synthesis triggered S1P synthesis in the glia, which was transported to neurons, leading to inflammation and neurodegeneration.^54^ RNAi targeting *CDase* and *SK1* (fly ortholog of *SPHK1*) led to improved *Drosophila* eclosion, while RNAi targeting *SK2* (fly ortholog of *SPHK2*) did not have any effect,^54^ suggesting it is the *SPHK1*-mediated synthesis of S1P that leads to neurodegeneration. This particular mechanism of inflammation and neurodegeneration from S1P overload via VLCFAs may be specific to certain cells of the nervous system and not necessarily present in other cells or tissue types, which would explain why *SPHK1* expression was only elevated in FRDA human PSNs.

Despite *SPHK1* expression already being high, treatment with K6PC-5 further enhanced *FXN* mRNA and protein levels. XY-14 treatment in FRDA PSNs also resulted in significantly increased frataxin protein expression. Therefore, it is reasonable to propose that XY-14 reduces Cer accumulation by targeting *LPP* enzymes involved in the metabolism of both C1P and S1P. Through this dual mechanism, XY-14 may be more effective than K6PC-5, which only acts on *SPHK*. The effect of K6PC-5 on frataxin expression may be mediated through *Nrf2*, as in the condition where *Nrf2* expression is low, *SPHK1* expression may be compensatorily upregulated by the sphingolipid rheostat to counteract oxidative stress, consistent with previously reported observations in SH-SY5Y cells.^28^ K6PC-5 significantly increased both *Nrf2* mRNA and protein expression in PSNs, supporting the hypothesis that *Nrf2* may bind to AREs upstream of the *FXN* TSS, a mechanism that could be operative in these neurons. In contrast, although XY-14 did not significantly affect *Nrf2* expression, it still resulted in a substantial increase in frataxin protein levels, which suggests that XY-14’s effect is more related to its role in C1P and LPA levels rather than a direct action on *Nrf2*. Although only K6PC-5 significantly affected ROS levels, both compounds induced a marked increase in frataxin expression. This finding is particularly important, given that profound frataxin deficiency is a defining hallmark of FRDA.

It is important to note that results identified *in vitro* are not necessarily reflective of outcomes *in vivo*. To address this, an *in vivo* validation study was designed and conducted over a 4-day period to assess the safety and efficacy of K6PC-5 and XY-14. Both 0.05 mg/kg/d K6PC-5 and 0.1 mg/kg/d XY-14 were well tolerated when administered intraperitoneally. In YG8sR mice, treatment with both compounds significantly increased Nrf2 expression, while XY-14 also significantly increased aconitase activity, indicating a positive effect on mitochondrial function. Targeting *Sphk* and *Lpp* led to moderate improvements in frataxin expression, although it is important to emphasize that this study was a short-term pilot study primarily aimed at confirming safety. Longer-term treatment may be required to achieve more substantial improvements in frataxin levels and mitochondrial function. Overall, these findings support the potential of both compounds for further investigation as therapeutic strategies for FRDA. Further studies with extended treatment duration and additional functional assessments will be crucial to determine their full efficacy and impact on FRDA disease progression *in vivo*.

In conclusion, this study identified dysregulation of sphingolipid-metabolizing enzymes in FRDA, particularly *SPHK* and *LPP*, across various models. Targeting these enzymes both *in vitro* and *in vivo* led to notable improvements in mitochondrial function and frataxin expression, highlighting the sphingolipid rheostat as a promising therapeutic target for FRDA.

### Limitations of the study

Our study has several limitations. Firstly, we did not perform any metabolomics experiment to specifically identify which sphingolipids are affected by treatments in either the human FRDA fibroblasts or iPSC-derived PSNs. However, we confirmed that the compounds exert the expected effects on the enzymes regulating these sphingolipids. Secondly, our knockdown using shRNA led to a moderate reduction in *LPP1* expression rather than a complete knockdown. Therefore, the effect of a full knockdown of *LPP1* remain unclear. Future studies could explore complete knockout models in both human FRDA fibroblasts and PSNs to assess potential adverse outcomes. Thirdly, only two pairs of isogenic PSNs were used, limiting sample size. While a larger sample size would provide more robust data, the use of isogenic pairs ensures that the observed differences are attributable to the repeat expansion and not to the genetic background. In addition, while both K6PC-5 and XY-14 improved various disease-associated characteristics across multiple models, some differences were observed between compounds and cell types. These results suggest that future studies should evaluate both compounds in parallel to determine the optimal therapeutic strategy. Our *in vivo* study was also short-term; therefore, long-term efficacy studies incorporating functional assessments, such as motor coordination, sensory function, and mitochondrial activity, are needed to fully evaluate sustained therapeutic benefits and disease modification. Finally, although donor sex is reported for all fibroblast and iPSC lines, the number of lines included within individual experimental groups was not sufficient to support meaningful evaluation of sex- or gender-related effects on experimental outcomes.

## Resource availability

### Lead contact

Further information and requests for resources and reagents should be directed to and will be fulfilled by the lead contact, Sara Anjomani Virmouni (sara.anjomanivirmouni@brunel.ac.uk).

### Materials availability

Materials established in this study are available from the lead contact upon request.

### Data and code availability

• All data reported in this paper will be shared by the lead contact upon request.
• This paper does not report original code.
• Any additional information required to reanalyze the data reported in this paper is available from the lead contact upon request.

## Acknowledgments

This work was supported by funding from the 10.13039/100002108Friedreich's Ataxia Research Alliance (10.13039/100002108FARA), 10.13039/501100000346Ataxia UK, the Association Française Ataxie de Friedreich (AFAF), and 10.13039/100014865the Foundation for Rare Diseases. The iPSCs were obtained from the Friedreich’s Ataxia Cell Line Repository (FACLR), established by the Napierala Laboratory at the 10.13039/100008333University of Alabama at Birmingham (10.13039/501100011104UAB) in collaboration with Dr. David Lynch from the 10.13039/100006458Children’s Hospital of Philadelphia (10.13039/100006458CHOP). N.C.M. is funded by a 10.13039/501100000291Kidney Research UK PhD Studentship (ST_009_20210728) awarded to Barbara Tanos. We also gratefully acknowledge the technical team at the Bio-Annexe facilities at Brunel University of London for their valuable support and assistance throughout this work.

## Author contributions

Z.R., methodology, validation, formal analysis, investigation, visualization, and writing – original draft; E.K.-E., validation, formal analysis, and investigation; S. Suleman, methodology, formal analysis, and investigation; F.J.E., formal analysis and investigation; S. Szunyogh, resources and methodology. O.G., investigation. N.C.M., methodology; A.V., investigation; R.W.-M., resources and methodology; C.P., resources and methodology; S.A.V., conceptualization, resources, methodology, formal analysis, investigation, writing – review and editing, funding acquisition, project administration, and supervision.

## Declaration of interests

The authors declare that they have no known competing financial interests or personal relationships that could have appeared to influence the work reported in this paper.

## STAR★Methods

### Key resources table

**Table:** 

REAGENT or RESOURCE	SOURCE	IDENTIFIER
**Antibodies**		

Anti-Frataxin antibody	Abcam	#ab175402; RRID: AB_2935904
Anti-Tubulin antibody	Abcam	#ab6160; RRID: AB_305328
Anti-SPHK1 antibody	Santa Cruz Biotechnology	#sc-365401; RRID: AB_10859210
Anti-PAP-2a Antibody	Santa Cruz Biotechnology	#sc-515517; RRID: N/A
Anti-Peripherin IgG2_a_κ	Santa Cruz Biotechnology	#sc-377093; RRID: AB_2923264
Anti-Brn3a IgG2_b_κ	Santa Cruz Biotechnology	#sc-8429; RRID: 626765
Goat Anti-Rabbit	Dako, Agilent Technologies	#P044801-2; RRID: AB_2617138
Goat Anti-Mouse	Dako, Agilent Technologies	#P044701-2; RRID: AB_2617137
Rabbit Anti-Rat	Abcam	#ab6734; RRID: AB_955450
Goat Anti-Mouse IgG2_a_ Alexa Fluor™ 594	ThermoFisher™ Scientific	#A-21135; RRID: AB_2535774
Goat Anti-Mouse IgG2_b_ Alexa Fluor™ 488	ThermoFisher™ Scientific	#A-21141; RRID: AB_2535778

**Recombinant DNA**		

pCMVR8.74	Gifted by Professor Michael Themis, Brunel University of London	-
pMD2.G	Gifted by Professor Michael Themis, Brunel University of London	-
SMARTvector lentiviral PLPP1 hCMV-TurboGFP: Empty Control Vector	Horizon Discovery Ltd	-
SMARTvector lentiviral PLPP1 hCMV-TurboGFP: shRNA (LPP1 6181)	Horizon Discovery Ltd	-

**Experimental models: Cell lines**		

Healthy donor fibroblast cell line H-Normal	Dr Terry Roberts (Brunel University of London)	N/A
Healthy donor fibroblast cell lines GM07525, GM07492, GM07545, GM08333, GM08399, GM23971, GM23976	Coriell Cell Repository (NJ, USA)	N/A
FRDA patient cell lines FA1, FA2, FA3, FA4	Dr Aurélien Bayot (Université Paris, France)	N/A
FRDA patient cell lines GM03816, GM03665, GM04078	Coriell Cell Repository (NJ, USA)	N/A
HEK293T cell lines	-	-
Control and FRDA iPSCs	Friedreich’s Ataxia Cell Line Repository (FACLR) established by Dr Marek Napierala and Dr David Lynch	Control: 3.5T, 59H FRDA: 7T, 59 NB 3.5T is the isogenic pair of 7T; 59H is the isogenic pair of 59

**Experimental models: Organisms/strains**		

Control and FRDA humanised transgenic mouse models	The Jackson Laboratory	Control: Y47R (#024097), FRDA: YG8sR (#024113)

**Chemical, peptides, and recombinant proteins**		

K6PC-5	Sigma-Aldrich	#SML1709
XY-14	Echelon Biosciences	#L-9218
4x Laemmli Sample Buffer	BioRad	#161-0747
10x TGS Buffer	BioRad	#161-0732
β-Mercaptoethanol	Sigma-Aldrich	#M-3148
Accutase	Fisher Scientific	#12780000
B-27™ Supplement	Fisher Scientific	#11500446
Bovine Serum Albumin (BSA)	Sigma-Aldrich	#A2153-50G
BrainPhys™ Neuronal Medium	Stem Cell Technologies	#05790
CellAdhere™ Dilution Buffer	Stem Cell Technologies	#07183
Cell Lysis Buffer 10X	Cell Signaling Technology	#9803
Chloroform	Sigma-Aldrich	#C2432-1L
Clarity Western ECL Substrate	BioRad	#1705061
CM-H2-DCFDA	ThermoFisher™ Scientific	#C6827
Cytosense LI™ (CY6)	Gifted by Dr Charareh Pourzand, University of Bath	-
DAPI	ThermoFisher™ Scientific	#62248
Deferoxamine Mesylate (DFO)	Sigma-Aldrich	#D9533
DMEM w/ Glutamax & Pyruvate	Fisher Scientific	#11594446
DMSO	Fisher Scientific	#10499683
Doxycycline hyclate	Sigma-Aldrich	#D9891
DPBS, no calcium, no magnesium	Fisher Scientific	#15326239
DPBS, calcium, magnesium	Fisher Scientific	#13492609
Dual Color Standard	BioRad	#161-0394
Ethanol	Fisher Scientific	#10542382
Fast SYBR™ Green Master Mix	Fisher Scientific	#10459604
Fetal Bovine Serum	Fisher Scientific	#A5256701
HBSS	Fisher Scientific	#12549069
Human Recombinant BDNF	Stem Cell Technologies	#78005
Human Recombinant GDNF	Stem Cell Technologies	#78058
Human Recombinant NGF-beta	Stem Cell Technologies	#78092
Human Recombinant NT3	Stem Cell Technologies	#78074
Hydrogen Peroxide	Fisher Scientific	#10386643
Insulin-Transferrin-Selenium-Sodium Pyruvate (ITS-A)	Fisher Scientific	#12067589
Isopropanol	Fisher Scientific	#10674732
Laminin Mouse Protein, Natural	ThermoFisher™ Scientific	#23017015
Mitosense LI™ (M)	Gifted by Dr Charareh Pourzand, University of Bath	-
MitoSOX™ Mitochondrial Superoxide Indicator	Fisher Scientific	#11579096
MitoTracker™ Green FM Dye	Fisher Scientific	#17501655
N-2 Supplement	Fisher Scientific	#11520536
N2 Supplement-A	Stem Cell Technologies	#07152
Neurobasal™ Medium	Fisher Scientific	#11570556
NeuroCult SM1 Neuronal Supplement	Stem Cell Technologies	#05711
Oligonucleotides	Sigma-Aldrich	#OLIGO
Paraformaldehyde	ThermoFisher™ Scientific	#047340-9M
Penicillin-Streptomycin	Fisher Scientific	#11528876
PMSF	Cell Signaling Technology	#8553
Ponceau S solution	Sigma-Aldrich	#P7170-1L
PrestoBlue™ Cell Viability Reagent	ThermoFisher™ Scientific	#A13261
ProLong™ Gold Antifade Mountant	ThermoFisher™ Scientific	#P36934
Puromycin Dihydrochloride	Sigma-Aldrich	#P8833
RNase/DNase-free dH_2_O	Fisher Scientific	#12060346
SKIM MILK POWDER	OXOID	#LP0031
STEMdiff™ Neural Crest Differentiation Kit	Stem Cell Technologies	#08610
Surface Decontaminant RNAse AWAY	Fisher Scientific	#10666421
TBS, Tris Buffered Saline, 10X Solution	Fisher Scientific	#10776834
TeSR™-E8™	Stem Cell Technologies	#05990
TRIzol™ Reagent	Fisher Scientific	#15596026
TRYPSIN .05% EDTA	Fisher Scientific	#11590626
Tween™-20	Fisher Scientific	#10419000
Vitronectin XF™	Stem Cell Technologies	#07180
Y-27632 (Dihydrochloride)	Stem Cell Technologies	#72302

**Critical commercial assays**		

Aconitase Assay Kit	Cayman Chemical	#705502
Citrate Synthase Assay Kit	Sigma-Aldrich	#CS0720
DNeasy® Blood & Tissue Kit	QIAGEN	#69504
Glutathione Fluorescent Detection Kit	ThermoFisher™ Scientific	#EIAGSHF
Human Frataxin ELISA Kit	Abcam	#ab176112
Human Phospholipid Phosphatase 1 (PLPP1) ELISA Kit	Abbexa	#abx555858
Malachite Green Phosphate Detection Kit	Cell Signaling Technology	#12776
Pierce™ BCA Protein Assay Kit	ThermoFisher™ Scientific	#23225
Sphingosine Kinase Activity Assay	Echelon Biosciences	#K-3500
SPHK2 Colorimetric Cell-Based ELISA Kit	Assay Genie	#CBCAB00862
TMRM Assay Kit	Abcam	#ab228569
QuantiTect® Reverse Transcription Kit	QIAGEN	#205311

**Software and algorithms**		

GeneSys Image Acquisition Software	Syngene	-
Microsoft Excel	Microsoft Corporation	-
GraphPad Prism 10	GraphPad Software	-

**Other**		

ACEA Novocyte Flow Cytometer	Agilent Technologies	-
CLARIOstar® Microplate Reader	BMG LABTECH	-
Countess™ Automated Cell Counter	ThermoFisher™ Scientific	-
Criterion™ Vertical Electrophoresis Cell	BioRad	-
Eppendorf Micro Centrifuge Model 5415 R	Eppendorf	-
FLoid™ Cell Imaging Station	Fisher Scientific	-
G:Box Chemi XRQ	Syngene	-
Heracell™ VIOS 160i CO2 Incubator, 165 L	ThermoFisher™ Scientific	-
HF14 Leica DM4000	Leica Microsystems	-
NanoDrop™ 2000c Spectrophotometer	ThermoFisher™ Scientific	-
NovoCyte Flow Cytometer	Agilent	-
Peltier Thermal Cycler	MJ Research	
PowerPac™ Basic Power Supply	BioRad	-
QuantStudio™ 7 Flex Real-Time PCR System	ThermoFisher™ Scientific	-
Techne® Dri-Block® heater	Sigma-Aldrich	-
Trans-Blot® Turbo™ Transfer System	BioRad	-
xMark™ Microplate Absorbance Spectrophotometer	BioRad	-
4-20% Criterion™ TGX™ Precast Midi Protein Gel	BioRad	#5671094
Cell Counting Chamber Slide Countess	Fisher Scientific	#10399053
Corning™ UV-Transparent Microplates	Fisher Scientific	#10288521
Greiner CELLSTAR® 96 well plates	Sigma-Aldrich	#M0562
MicroAmp® Fast Optical 96-Well Reaction Plate	Fisher Scientific	#4346906
MicroAmp™ Optical Adhesive Film	Fisher Scientific	#4311971
Nunc™ Cell Culture 100x15mm Dish	Fisher Scientific	#10508921
Nunc™ Cell-Culture Treated Multidishes (6 Well)	Fisher Scientific	#10119831
Nunc™ Cell-Culture Treated Multidishes (12 Well)	Fisher Scientific	#10098870
Nunc™ Cell-Culture Treated Multidishes (24 Well)	Fisher Scientific	#10376912
Nunc™ MicroWell™ 96-Well Microplate	Fisher Scientific	#10212811
Nunc™ MicroWell™ 96-Well, Non-Treated, No Lid	Fisher Scientific	#11361585
Trans-Blot Turbo Transfer Pack	BioRad	#1704159

### Experimental model and study participant details

#### Primary human fibroblast cell lines

Human FRDA and control fibroblasts were grown Gibco™ Dulbecco’s Modified Eagle Medium (DMEM) GlutaMAX™ (Fisher Scientific) supplemented with 10% Fetal Bovine Serum (FBS) (Fisher Scientific) and 1% penicillin-streptomycin (Fisher Scientific). HEK293T cells were grown Gibco™ Dulbecco’s Modified Eagle Medium (DMEM) GlutaMAX™ (Fisher Scientific) supplemented with 10% Fetal Bovine Serum (FBS) (Fisher Scientific) and 1% penicillin-streptomycin (Fisher Scientific). In compound experiments, cell lines were treated with 0.1 μM – 200 μM K6PC-5, 0.1 μM – 10 μM XY-14 or vehicle (DMSO (Fisher Scientific), up to a final concentration of 0.1% v/v for all conditions) for 72 hours.

Fibroblasts from healthy donors GM07525 (Female, 22 years), GM07492 (Male, 17 years), GM07545 (Female, 22 years), GM08333 (Male, 5 months), GM08399 (Female, 19 years), GM23971 (Male, 33 years) and GM23976 (Male, 22 years) were obtained from the Coriell Cell Repository (Camden, NJ, USA), and H-Normal (Male, 27 years) from Dr. Terry Roberts (Brunel University of London, UK) with informed consent. Fibroblast from FRDA patients GM04078 (Male, 30 years, GAA 541/420), GM03816 (Female, 36 years, GAA 330/380) and GM03665 (Female, 13 years, GAA 445/740) were obtained from the Coriell Cell Repository (Camden, NJ, USA). Fibroblasts from FRDA patients FA-1 (Female, 8 years, GAA 460/700), FA-2 (Female, 11 years, GAA 610/770), FA-3 (Female, 14 years, GAA 780/780) and FA-4 (Female, 8 years, GAA 790/970) were provided by Dr. Aurélien Bayot (Université Paris, France); informed consent was obtained from patients and/or legal guardians according to protocols approved by the Robert Debré Hospital Ethical Committee (Paris, France). All samples were fully de-identified prior to transfer.

#### Human iPSC-derived cell lines

Four iPSC lines, two FRDA and two isogenic controls, were obtained from the FACLR operated by Dr. Marek Napierala (UT Southwestern Medical Center) and Dr. David Lynch (Children’s Hospital of Philadelphia). The FRDA iPSC lines used were 7T (Female, 28 years, GAA 830/830) and 59 (Male, 37 years, GAA 550/830). Their corresponding isogenic control lines were 3.5T (isogenic to 7T) and 59H (isogenic to 59). Primary fibroblast samples used to generate these iPSC lines were collected under ethical approvals from the Children’s Hospital of Philadelphia (IRB 10-007864) and UT Southwestern Medical Center (IRB STU-2023-0478). Written informed consent was obtained from all donors and/or legal guardians, and all samples were fully de-identified prior to distribution. iPSC lines were generated and banked under UT Southwestern IRB-approved protocols and informed consent.

Human FRDA and control iPSCs were grown in TeSR™-E8™ media (Stem Cell Technologies) in cultureware coated with Vitronectin XF™ (Stem Cell Technologies) in CellAdhere™ Dilution Buffer (Stem Cell Technologies) to a final concentration of 10 μg/mL. Cells were supplemented with 10 μg/mL Y-27632 (Stem Cell Technologies) for 24 hours following thawing or passaging.

For CM differentiation, iPSCs were differentiated using a defined protocol,^55^ and subsequently dissociated using the STEMdiff™ Cardiomyocyte Dissociation Kit (Stem Cell Technologies) prior to harvesting. iPSCs and iPSC-derived CMs were characterised by qRT-PCR (Figure S3). iPSCs showed significantly higher expression of pluripotency markers *OCT4* (Figure S3A) and *NANOG* (Figure S4B) compared to CMs. Conversely, CMs markers *MYH7* (Figure S3C) and *cTNNI* (Figure S3D) were significantly upregulated in differentiated cells, confirming successful differentiation. For PSN differentiation, iPSCs were cultured on Laminin-coated (ThermoFisher) plates prepared in DPBS containing calcium and magnesium (Fisher Scientific), with a final concentration of 10 μg/mL. iPSCs were differentiated to neural crest cells using the STEMdiff™ Neural Crest Differentiation Kit (Stem Cell Technologies). Neural crest cells were differentiated to PSNs using Gibco™ Neurobasal™ Medium (Fisher Scientific) and BrainPhys™ Neuronal Medium (Stem Cell Technologies), supplemented with 2 μg/mL doxycycline (for the first three days of differentiation), 10 μg/mL Y-27632, 10 ng/mL BDNF, 10 ng/mL GDNF, 10 ng/mL NT3, and 10 ng/mL β-NGF (all from Stem Cell Technologies). HEK cell lines were used for transfections of lentiviruses containing *LPP1* shRNA constructs. All cell lines were incubated at 37°C and 5% CO_2_.

Donor sex is reported for all lines. The number of lines within individual experimental groups was insufficient to assess sex- or gender-related effects on experimental outcomes.

#### Transgenic mice

Housing and husbandry of Y47R and YG8sR mice were maintained under standard laboratory conditions, including controlled temperature, humidity, and a 12-hour light/dark cycle, with free access to food and water.^56^ All procedures were carried out in accordance with the UK Home Office ‘*Animals* (*Scientific Procedures*) *Act 1986*’ and approved by the Brunel University Animals Welfare and Ethical Review Board (AWERB). Mice were genotyped to confirm Y47R and YG8sR status, and GAA repeat expansion and *FXN* expression were verified prior to experimental use.^56^^,^^57^^,^^58^^,^^59^^,^^60^^,^^61^

### Method details

#### Compounds

K6PC-5, a potent agonist which selectively targets *SPHK1*, was purchased from Sigma-Aldrich. XY-14, a competitive inhibitor of *LPP1*, was purchased from Echelon Biosciences.

#### shRNA-mediated reduction of LPP1

SMARTvector lentiviral PLPP1 hCMV-TurboGFP shRNA constructs (Empty Vector and LPP1 6181) were purchased from Horizon Discovery Ltd and amplified in DH5alpha *E. coli* cells. The pCMVR8.74 and pMD2.G constructs were kindly provided by Professor Michael Themis and propagated in DH5alpha cells. Lentivirus was produced using Genejuice® transfection reagent according to the manufacturer’s instructions.^62^ Sucessful transfection for lentiviral production was confirmed by assessing GFP expression and syncytia formation. Viral supernatant was incubated in complete cell culture medium containing 5 μg/ml polybrene for 20 min prior to infection of HEK293T cells. Cells were imaged 72 hours post-transduction and analysed for GFP expression.

#### RNA isolation and quantitative real time polymerase chain reaction (qRT-PCR)

Total RNA was isolated from human FRDA and control fibroblast cell lines or Y47R and YG8R mouse tissue using TRIzol™ Reagent (Fisher Scientific). Up to 1 μg of RNA was used to synthesis cDNA using the QuantiTect Reverse Transcription Kit (QIAGEN). qRT-PCR was completed using the QuantStudio Flex Real-Time PCR Machine and SYBR® Green (Fisher Scientific). The primers which were used are summarised in Table S1. All primers were purchased from Sigma-Aldrich. Experiments were also completed using cells following treatment with 10 μM K6PC-5, 0.1 μM XY-14, 1 μM XY-14 or Vehicle for 72 hours. qRT-PCRs were completed in triplicate in a minimum of two independent experiments.

#### SPHK2 protein quantification by ELISA

Protein expression of *SPHK2* was investigated using the Human Sphingosine Kinase 2 (SPHK2) ELISA Kit (Assay Genie), as per the manufacturer’s instructions. Cells were seeded in the provided 96-well plate, with 48 wells kept for *SPHK2* measurement and 48 wells kept for *GAPDH* measurement for normalisation. The absorbance at 450 nm was measured for all standards and samples using the xMark Microplate Absorbance Spectrophotometer (BioRad) and protein expression interpolated from the standard curve generated.

#### SPHK1/2 enzymatic activity

Enzymatic activity of *SPHK1*/*2* was confirmed using the Sphingosine Kinase Activity Assay (Echelon Biosciences), as per the manufacturer’s instructions. Cell lysates were prepared by resuspending cells in the Reaction Buffer provided and freeze-thawing 3 times before centrifugation, supernatant collected, and protein concentrations calculated using the Pierce™ BCA Protein Assay Kit (ThermoFisher™ Scientific). Luminescence (RLU) was measured for all standards and samples using the CLARIOstar® Microplate Reader and protein expression interpolated from the standard curve generated. Luminescence generated is inversely proportional to *SPHK1*/*2* activity, therefore values were transformed to convey *SPHK1*/*2* enzymatic activity more clearly.

#### LPP1 protein quantification by ELISA

Protein expression of *LPP1* was validated using the Human Phospholipid Phosphatase 1 (PLPP1) ELISA Kit (Abbexa), as per the manufacturer’s instructions. Cell lysates were prepared by resuspending cells in PBS and freeze-thawing 3 times before centrifugation, supernatant collected, and protein concentrations calculated using the Pierce™ BCA Protein Assay Kit (ThermoFisher™ Scientific). The absorbance at 450 nm was measured for all standards and samples using the xMark Microplate Absorbance Spectrophotometer (BioRad) and protein expression interpolated from the standard curve generated.

#### Total phosphatase activity

Total phosphatase activity was measured using the Malachite Green Phosphate Detection Kit (Cell Signaling Technology). Cell lysates were prepared by resuspending cells in PBS and freeze-thawing 3 times before centrifugation, supernatant collected, and protein concentrations calculated using the Pierce™ BCA Protein Assay Kit (ThermoFisher™ Scientific). The absorbance at 630 nm was measured for all standards and samples using the xMark Microplate Absorbance Spectrophotometer (BioRad) and protein expression interpolated from the standard curve generated.

#### PrestoBlue® cell viability assay

Cells were seeded for 24 hours prior to treatment with 0.1 μM – 200 μM K6PC-5, 0.1 μM – 10 μM XY-14 or vehicle for 72 hours in triplicate. Following treatment, PrestoBlue® Cell Viability Reagent (ThermoFisher™ Scientific) was added to the cells to make up a final concentration of 1X PrestoBlue® and the cells incubated for 3 hours. The absorbances at 570 nm and 600 nm were measured using the xMark Microplate Absorbance Spectrophotometer (BioRad). The absorbance values at 600 nm were used to normalise the values obtained at 570 nm, as per the manufacturer’s instructions.

#### ROS measurement

Flow cytometry was used to determine levels of mROS and tROS. Cells were seeded for 24 hours prior to treatment with 10 μM K6PC-5, 0.1 μM XY-14, 1 μM XY-14 or vehicle for 72 hours. Cells were then incubated in 5 μM of MitoSOX™ Red Mitochondrial Superoxide Indicator (Fisher Scientific) for 30 minutes for determining mROS, or 20 μM CM-H2DCFDA (Fisher Scientific) for 45 minutes for determining tROS. Cells were washed and resuspended in HBSS (Fisher Scientific) before flow cytometry analysis. For mROS, fluorescence was visualised at Ex/Em = 396/610 nm and for tROS fluorescence was visualised at Ex/Em = 492/527 nm.

#### Aconitase activity assay

Aconitase activity was quantified using the Aconitase Assay Kit (Cayman Chemical). Cells were seeded for 24 hours prior to treatment with 10 μM K6PC-5, 0.1 μM XY-14, 1 μM XY-14 or vehicle for 72 hours. Protein lysates were extracted using 1X Cell Lysis buffer (Cell Signaling Technology) and quantified using the Pierce™ BCA Protein Assay Kit (ThermoFisher™ Scientific). 500 μg/mL of protein lysate per cell type and condition was loaded onto the provided 96-well plate according to the manufacturer’s instructions. The absorbance at 340 nm was measured for all standards and samples using the xMark Microplate Absorbance Spectrophotometer (BioRad) and expression interpolated from the standard curve generated. Results were normalised to citrate synthase activity. Citrate synthase activity was quantified using the Citrate Synthase Assay Kit (Merck). 20 μg/mL of each sample used in the Aconitase Assay Kit was loaded onto a 96-well plate according to the manufacturer’s instructions. The absorbance at 412 nm was measured for all standards and samples once every minute for 15 minutes using the xMark Microplate Absorbance Spectrophotometer (BioRad). Following this, 10mM oxaloacetic acid was added to all wells and the absorbance at 412 nm was measured for all standards and samples once every minute for 30 minutes using the xMark Microplate Absorbance Spectrophotometer (BioRad), and expression interpolated from the standard curve generated.

#### Mitochondrial membrane potential (ΔΨm)

Mitochondrial membrane potential was determined using the TMRM Assay Kit (Mitochondrial Membrane Potential) (Abcam). Cells were seeded for 24 hours prior to treatment with 10 μM K6PC-5, 0.1 μM XY-14, 1 μM XY-14 or Vehicle for 72 hours. Cells were then incubated in 50 nM of TMRM reagent for 30 minutes. Fluorescence was visualised at Ex/Em = 488/575 nm.

#### Mitochondrial mass

Flow cytometry was used to determine mitochondrial mass. Cells were seeded for 24 hours prior to treatment with 10 μM K6PC-5, 0.1 μM XY-14, 1 μM XY-14 or Vehicle for 72 hours. Cells were then incubated in 250 nM of MitoTracker™ Green FM Dye (Fisher Scientific) for 15 minutes. Cells were washed and resuspended in HBSS before flow cytometry analysis. Fluorescence was visualised at Ex/Em = 490/516 nm.

#### Mitochondrial DNA copy number

DNA was isolated from cells treated with 10 μM K6PC-5, 0.1 μM XY-14, 1 μM XY-14 or Vehicle for 72 hours using the DNeasy Blood & Tissue Kit (QIAGEN). Mitochondrial copy number was then detected using qPCR, using the QuantStudio Flex Real-Time PCR Machine and SYBR® Green (Fisher Scientific). The primers which were used are summarised in Table S1. All primers were purchased from Sigma-Aldrich. qPCRs were completed in triplicate in a minimum of two independent experiments.

#### H_2_O_2_-induced cell viability assay

Cells were seeded for 24 hours prior to treatment with 0.1 μM – 200 μM K6PC-5, 0.1 μM – 10 μM XY-14 or vehicle for 72 hours in triplicate. Following this, cells were then treated with 100 μM H_2_O_2_ in serum-free media for 1 hour. The H_2_O_2_ was then removed and replaced with complete media for 24 hours. PrestoBlue® Cell Viability Assay was then carried out as mentioned previously.

#### Glutathione assay

GSH/GSGG was determined using the Glutathione Fluorescent Detection Kit (Invitrogen™). Cells were seeded for 24 hours prior to treatment with 10 μM K6PC-5, 0.1 μM XY-14, 1 μM XY-14 or vehicle for 72 hours. Cell lysates were prepared by resuspending cells in 1X Cell Lysis Buffer (Cell Signaling Technology) and freeze-thawing 3 times before centrifugation, supernatant collected, and protein concentrations calculated using the Pierce™ BCA Protein Assay Kit (ThermoFisher™ Scientific). Free GSH signal was measured initially using the CLARIOstar® Microplate Reader at Ex/Em 390/510 nm. The total GSH signal was then measured using the CLARIOstar® Microplate Reader at Ex/Em 390/510 nm and GSH/GSSG calculated according to the manufacturer’s instructions.

#### Frataxin protein quantification by ELISA

Frataxin protein expression of human FRDA and control fibroblast cell lines, iPSC-derived CMs, iPSC-derived PSNs, and shRNA-treated fibroblast cell lines was quantified using the Human Frataxin ELISA Kit (Abcam). For cells requiring treatment, cells were seeded for 24 hours prior to treatment with 10 μM K6PC-5, 0.1 μM XY-14, 1 μM XY-14 or Vehicle for 72 hours. ShRNA-treated cells were selected using 1 μg/mL puromycin for 24 hours prior to the experiment. Protein concentration of lysates was determined using the Pierce™ BCA Protein Assay Kit (ThermoFisher™ Scientific). The absorbance at 450 nm was measured for all standards and samples using the xMark Microplate Absorbance Spectrophotometer (BioRad) and expression interpolated from the standard curve generated.

#### Nrf2 quantification by ELISA

Nrf2 protein expression of human FRDA and control fibroblast cell lines, iPSC-derived CMs, iPSC-derived PSNs, and shRNA-treated fibroblast cell lines was quantified using the Human Nrf2 ELISA Kit (Abcam). For cells requiring treatment, cells were seeded for 24 hours prior to treatment with 10 μM K6PC-5, 0.1 μM XY-14, 1 μM XY-14 or vehicle for 72 hours. shRNA-treated cells were selected using 1 μg/mL puromycin for 24 hours prior to the experiment. Protein concentration of lysates was determined using the Pierce™ BCA Protein Assay Kit (ThermoFisher™ Scientific). The absorbance at 450 nm was measured for all standards and samples using the xMark Microplate Absorbance Spectrophotometer (BioRad) and expression interpolated from the standard curve generated.

#### Western blotting

Protein was extracted from human FRDA and control fibroblast cell lines, or Y47R/YG8sR mouse tissues using Cell Lysis Buffer (Cell Signaling Technology) supplemented with 1 mM PMSF (Cell Signaling Technology). Protein was quantified using the Pierce™ BCA Protein Assay Kit (ThermoFisher™ Scientific). Protein was diluted in 4x Laemmli Buffer (BioRad) to a final concentration of 30 μg per lysate. Lysates were run on 4-20% Criterion™ TGX™ Precast Midi Protein Gels (BioRad) and transferred to membranes using the Trans-Blot® Turbo™ Transfer System (BioRad). Ponceau S Solution (Sigma Aldrich) was used to confirm complete transfer of protein. Membranes were blocked in 5% milk in TBST (0.1% Tween-20). Membranes were incubated in primary antibody diluted in 5% BSA in TBST overnight at 4°C, followed by incubation in secondary antibody diluted in 2.5% milk in TBST for 1 hour at room temperature. Bands were visualised using Clarity Western ECL Substrate (BioRad) using the G:Box Chemi XRQ (Syngene). Experiments were completed for Y47R and YG8sR mouse tissues as well as untreated human FRDA and control fibroblasts or treated with 10 μM K6PC-5, 0.1 μM XY-14, 1 μM XY-14 or vehicle for 72 hours. The following antibodies were used: Anti-Frataxin antibody (1:500, Abcam), Anti-Tubulin antibody (1:1000, Abcam), Anti-SPHK1 Antibody (1:1000, Santa Cruz Biotechnology), Anti-PAP-2a Antibody (1:1000, Santa Cruz Biotechnology), Goat Anti-Rabbit Antibody (1:2000, Dako, Agilent Technologies), Goat Anti-Mouse Antibody (1:2000, Dako, Agilent Technologies), Rabbit Anti-Rat Antibody (1:2000, Abcam).

#### Cytosolic labile iron pool (cLIP)

Cells were seeded for 24 hours prior to treatment with 10 μM K6PC-5, 1 μM XY-14 or Vehicle for 72 hours. 70 μM Cytosense LI™ (CY6) was used to detect fluorescence and 100 μM deferoxamine mesylate (DFO) was used as an iron chelator. Cells were either kept untreated, treated with CY6, or treated with CY6 + DFO and incubated for 2 hours. Cells were then washed, resuspended in Fixing Buffer solution [10mM HEPES + 150mM NaCl], and fluorescence was visualised at Ex/Em = 435/473 nm. The difference in fluorescence in CY6 + DFO and CY6 treated cells was quantified for analysis of cLIP.

#### Mitochondrial labile iron pool (mLIP)

Cells were seeded for 24 hours prior to treatment with 10 μM K6PC-5, 1 μM XY-14 or Vehicle for 72 hours. 50 μM Mitosense LI™ (M) was used to detect fluorescence and 100 μM DFO was used as an iron chelator. Cells were either kept untreated, treated with M, or treated with M + DFO and incubated for 2 hours. Cells were then washed, resuspended in Fixing Buffer solution [10mM HEPES + 150mM NaCl], and fluorescence was visualised at Ex/Em = 330/550 nm. The difference in fluorescence in M + DFO and M treated cells was quantified for analysis of mLIP.

#### Immunofluorescence staining

iPSCs and iPSC-derived PSNs were plated on coverslips coated with Vitronectin XF and Laminin, respectively, 24 hours prior to fixing. Cells were fixed in PBS containing 4% formaldehyde followed by fixation in ice-cold methanol. Cells were permeabilised in PBST (0.1% Triton-X-100), blocked in 3% BSA in PBST, and incubated in primary antibody diluted in 3% BSA in PBST overnight at 4°C. Cells were then incubated in in secondary antibody diluted in 3% BSA in PBST for 1 hour at room temperature. DAPI was added to the cells for 5 minutes, followed by mounting of the coverslips onto slides using ProLong™ Gold Antifade Mountant (ThermoFisher™ Scientific). Cells were visualised using the HF14 Leica DM4000. Images were taken at both the iPSC stage as well as after differentiation to PSNs.

#### *In vivo* administration of K6PC-5 and XY-14 in YG8sR mice

A preliminary *in vivo* study was conducted to assess the safety and tissue effects of K6PC-5 and XY-14 in the YG8sR mouse model. Mice were genotyped to confirm either Y47R or YG8sR status and were age- and sex-matched (2-4 months old) prior to study initiation. K6PC-5, XY-14 or vehicle control (DMSO) was administered via i.p. injection once daily for four consecutive days. K6PC-5 was administered at a dose of 0.05 mg/kg/day, while XY-14 was administered at doses of 0.05 mg/kg/day or 0.1 mg/kg/day. At the end of the treatment period, mice were euthanised using a Schedule 1 method and tissues were collected. Cerebellar tissue was isolated for downstream molecular analyses.

### Quantification and statistical analysis

Results were calculated using Microsoft Excel and graphs were created using GraphPad Prism 10 (GraphPad Software). Quantification of Western blot results was carried out using FIJI. Results are expressed as mean ± standard error of the mean (SEM). Statistical significance was calculated using either unpaired Student’s *t*-test or two-way ANOVA, with significance set at *P* <0.05. Post-hoc tests were carried out following ANOVA. In the main article, Tukey’s multiple comparisons test used for all ANOVA except for Figure 3H, which used Holm-Šídák’s multiple comparisons test to allow specific comparison between untreated vs H_2_O_2_ + compound and H_2_O_2_ vs H_2_O_2_ + compound. In the supplementary, Figures S2, S5E, and S5F used Dunnett’s multiple comparisons test to compare each mean to untreated.
