# Supplementary material for: Dysregulation of sphingolipid-metabolizing enzymes in Friedreich’s ataxia: In vitro and in vivo insights into therapeutic targeting
Source: iScience. 2026 Jun 22;29(7):116479. doi: 10.1016/j.isci.2026.116479 (PMC13316217; doi:10.1016/j.isci.2026.116479)
Supplement: Document S1. Figures S1–S5, Table S1, and Data S1 [file mmc1.pdf]

iScience, Volume *29*

Supplemental information

Dysregulation of sphingolipid-metabolizing enzymes in Friedreich's ataxia: *In vitro* and *in vivo* insights into therapeutic targeting

Zenouska Ramchunder, Ester Kalef-Ezra, Saqlain Suleman, Fred Jonathan Edzeamey, Sandor Szunyogh, Owen Gittins, Natalia Castro Mena, Richard Wade-Martins, Adamo Valle, Charareh Pourzand, and Sara Anjomani Virmouni

Supplemental Information

Supplementary Table 1. List of primer sequences used for qPCR and qRT-PCR

**Primer Name**	**Sequence (5’ → 3’)**
**Human Primers**	
HPRT Forward	*5’-GGTGAAAAGGACCCCACGA-3’*
HPRT Reverse	*5’-TCAAGGGCATATCCTACAACA-3’*
FXN Forward	*5’-CAGACCAAACGCTGGACTCT-3’*
FXN Reverse	*5’-AGCCAGATTTGCTTGTTTGGC-3’*
SPHK1 Forward	*5’-CTTGCAGCTCTTCCGGACTC-3’*
SPHK1 Reverse	*5’-GCTCAGTGAGCATCAGCGTC-3’*
SPHK2 Forward	*5’-TTCTATTGGTCATCCCTTTGG-3’*
SPHK2 Reverse	*5’-AGCCCGTTCAGCACCTCA-3’*
LPP1 Forward	*5’-GTCGAGGGAATGCAGAAAGA-3’*
LPP1 Reverse	*5’-CCTTCATCCTGGCTTGAAGATA-3’*
LPP2 Forward	*5’-CCTACCGTCCAGATACCATCA-3’*
LPP2 Reverse	*5’-GTTGAAGTCCGAGCGAGAATAG-3’*
LPP3 Forward	*5’-CAAATCAGAAGGAGCCAGAGAA-3’*
LPP3 Reverse	*5’-CAGCAAGAGCAACTCCTACAA-3’*
MT-TL1 Forward	*5’-CACCCAAGAACAGGGTTTGT-3’*
MT-TL1 Reverse	*5’-TGGCCATGGGTATGTTGTTA-3’*
B2M Forward	*5’-TGCTGTCTCCATGTTTGATGTATCT-3’*
B2M Reverse	*5’-TCTCTGCTCCCCACCTCTAAGT-3’*
Nrf2 Forward	*5’-CACATCCAGTCAGAAACCAGTGG-3’*
Nrf2 Reverse	*5’-GGAATGTCTGCGCCAAAAGCTG-3’*
OCT4 Forward	*5’-CAATTTGCCAAGCTCCTGAAG-3’*

OCT4 Reverse	*5'- AAAGCGGCAGATGGTCGTT-3'*
NANOG Forward	*5'- CCTTCCTCCATGGATCTGCTT-3'*
NANOG Reverse	*5'- CTTGACCGGGACCTTGTCTTC-3'*
MYH7 Forward	*5'- CGTAGCGATCCTTGAGGTTGTA-3'*
MYH7 Reverse	*5'- CGTAGCGATCCTTGAGGTTGTA-3'*
cTNNI Forward	*5'- CCAACTACCGCGCTTATGC-3'*
cTNNI Reverse	*5'- CTCGCTCCAGCTCTTGCTTT-3'*
PRPH Forward	*5'- GAGGAGCTGCGACAGCTAAA-3'*
PRPH Reverse	*5'- ACCTCAGGCACAGTCGTCTT-3'*
Brn3a Forward	*5'- AGTACCCGTCGCTGCACTCCA-3'*
Brn3a Reverse	*5'- TTGCCCTGGGACACGGCGATG-3'*
**Mouse Primers**	
Gapdh Forward	*5'-ACCCAGAAGACTGTGGATGG-3'*
Gapdh Reverse	*5'-GGATGCAGGGATGATGTTCT-3'*
Fxn Forward	*5'-TTGAAGACCTTGCAGACAAG-3'*
Fxn Reverse	*5'-AGCCAGATTTGCTTGTTTGG-3'*
Sphk1 Forward	*5'-TCCTGGAGGAGGCAGAGAT-3'*
Sphk1 Reverse	*5'-GCTACACAGGGGTTTCTGGA-3'*
Sphk2 Forward	*5'-AAATCACCCCTGAATTGCTG-3'*
Sphk2 Reverse	*5'-ATGCCTTCCCACTCACTCAG-3'*
Lpp1 Forward	*5'-TGTACTGCATGCTGTTTGTCGCAC-3'*
Lpp1 Reverse	*5'-TGACGTCACTCCAGTGGTGTTTGT-3'*
Lpp2 Forward	*5'-TGGCCAAGTACATGATTGG-3'*
Lpp2 Reverse	*5'-AGCAGCCGTGCCCACTTCC-3'*
Lpp3 Forward	*5'-ATAAACGATGCTGTGCTCTGTGCG-3'*
Lpp3 Reverse	*5'-TTTGCTGTCTTCTCCTCTGCACCT-3'*

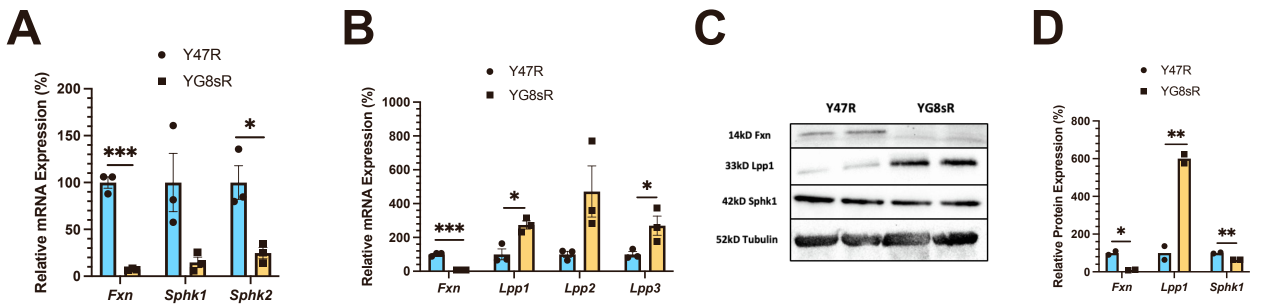

Supplementary Figure 1. Dysregulation of Sphk and Lpp in control and FRDA mouse tissue. qRT-PCR analysis of (A) *Sphk1/2* and (B) *Lpp1/2/3* gene expression in YG8sR mouse cerebellum tissue, normalised to Y47R line, n=3. (C) Representative immunoblots of Sphk1 and Lpp1 protein expression in YG8sR mouse cerebellum tissue, n=2. (D) Quantification of Sphk1 and Lpp1 protein expression measured by western blotting in YG8sR mouse cerebellum tissue, n=2. Data are represented as mean±SEM. statistical significance was determined using an unpaired, two-tailed Student's *t*-test (*P* <0.05. **P* <0.05, ***P* <0.01, ****P* <0.001).

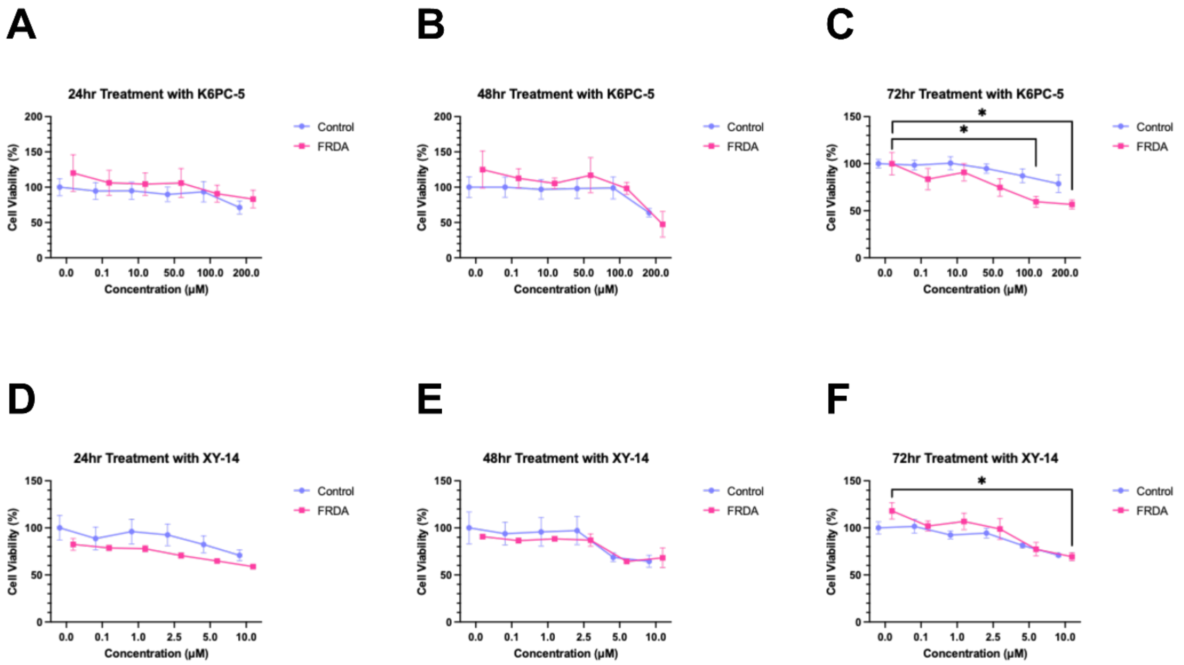

Supplementary Figure 2. Cell Viability assay to assess the safety and tolerability of K6PC-5 and XY-14 in human FRDA and control fibroblast cell lines. Cell viability in human FRDA and control fibroblast cell lines following treatment with K6PC-5 (0.1, 10, 50, 100 and 200 μM) was assessed at (A) 24 hours, (B) 48 hours and (C) 72 hours, and with XY-14 (0.1, 1, 2.5, 5 and 10 μM) at (D) 24 hours, (E) 48 hours and (F) 72 hours, using the PrestoBlue™ Cell Viability Reagent, n=2. Data are represented as mean±SEM. Statistical significance was determined using two-way ANOVA with Dunnett's multiple comparisons test (**P* <0.05).

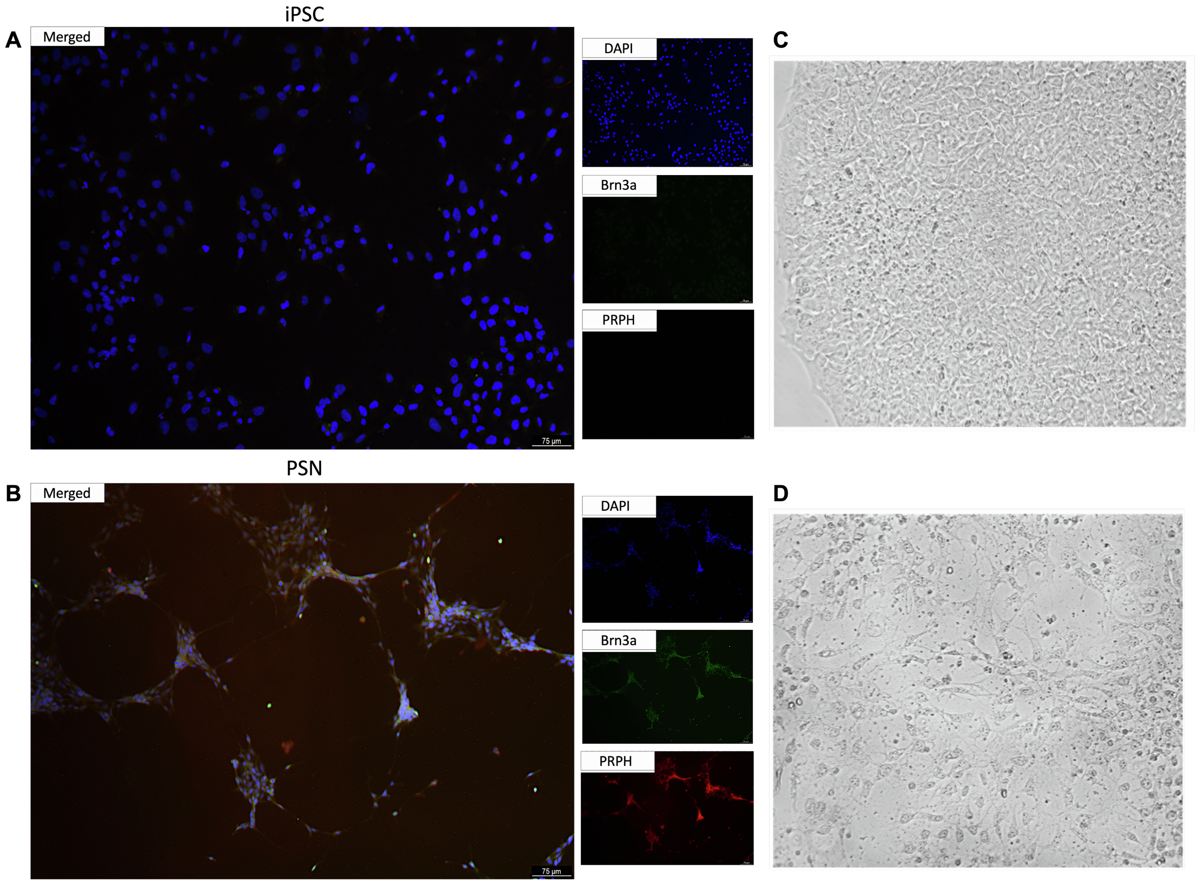

Supplementary Figure 3. Differentiation and characterisation of iPSC-derived peripheral sensory neurons (PSNs) using immunofluorescence (IF). Protein expression of *Brn3a* and *PRPH* was visualised in both iPSCs (A) and iPSC-derived PSNs (B) using the HF14 Leica DM4000. Images outlining the morphology of iPSCs (C) and PSNs (D) using the FLoid™ Cell Imaging station.

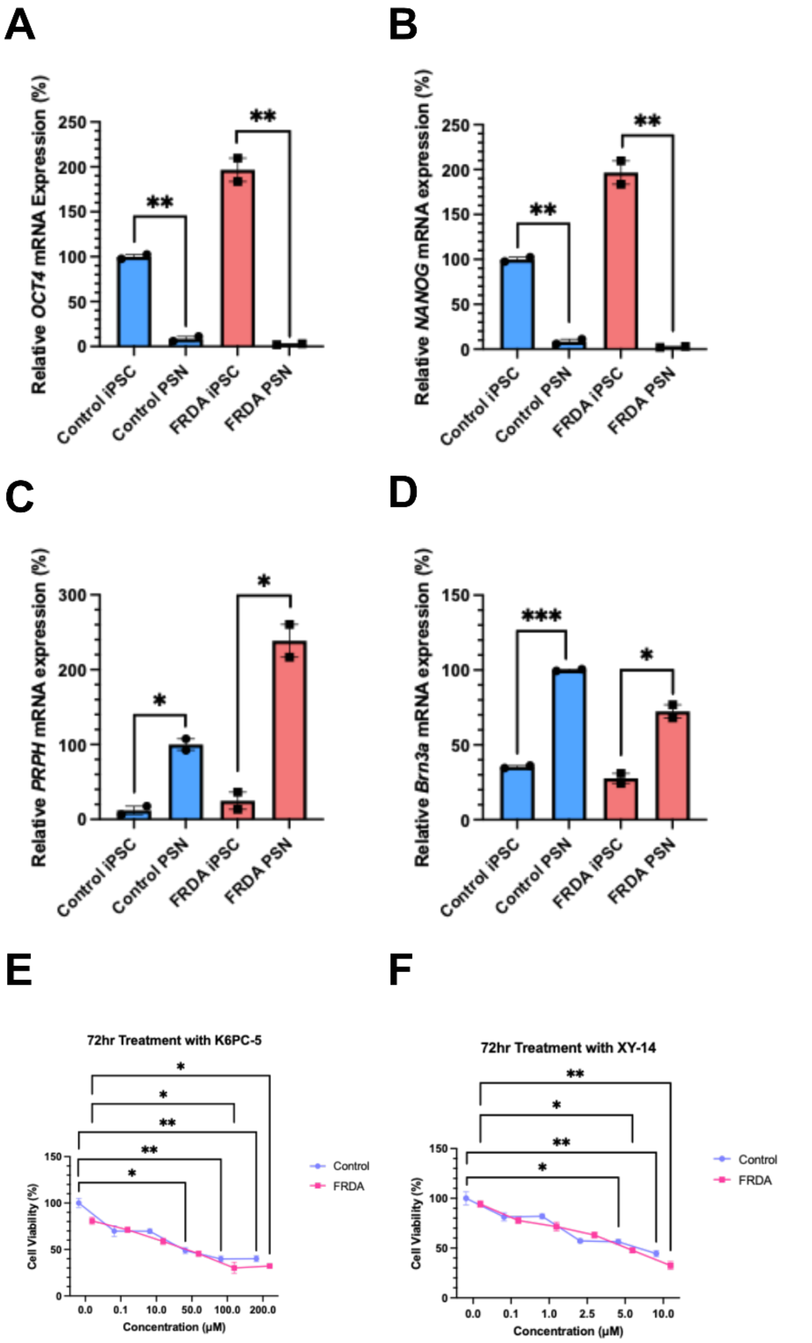

Supplementary Figure 4. qRT-PCR-based characterisation of iPSCs and iPSC-derived peripheral sensory neurons (PSNs) and compound toxicity assessment in human FRDA and control PSNs. qRT-PCR analysis of (A) *OCT4*, (B) *NANOG*, (C) *PRPH* and (D) *Brn3a* gene expression in control and FRDA PSNs compared to control and FRDA iPSCs, respectively, n=2. Cell viability in human FRDA and control PSNs following treatment with (E) K6PC-5 at 0.1, 10, 50, 100 and 200 µM, and (F) XY-14 at 0.1, 1, 2.5, 5 and 10 µM, assessed using the PrestoBlue™ Cell Viability Reagent at 72 hours, n=2. Data are represented as mean±SEM. statistical significance was determined using an unpaired, two-tailed Student's *t*-test or two-way ANOVA with Dunnett's multiple comparisons test (*P <0.05, **P <0.01).

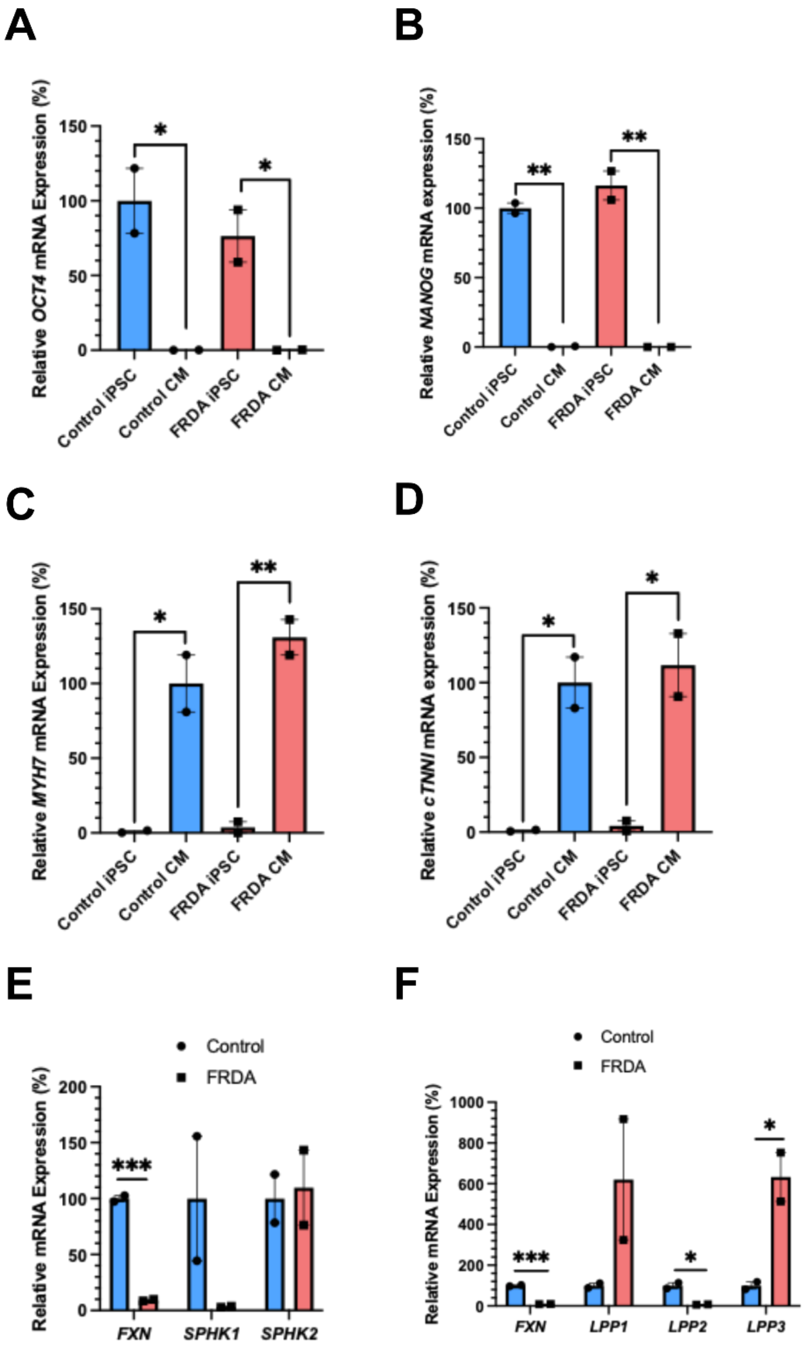

Supplementary Figure 5. qRT-PCR-based characterisation of iPSCs and iPSC-derived cardiomyocytes (CMs). qRT-PCR analysis of (A) *OCT4*, (B) *NANOG,* (C) *MYH7* and (D) *CTNNI* gene expression in control and FRDA CMs compared to control and FRDA iPSCs, respectively, n=2. qRT-PCR analysis of (E) *SPHK1/2* and (F) *LPP1/2/3* expression in human FRDA CMs, n=2. Data are represented as mean±SEM. statistical significance was determined using an unpaired, two-tailed Student's *t*-test (**P* <0.05, ***P* <0.01).

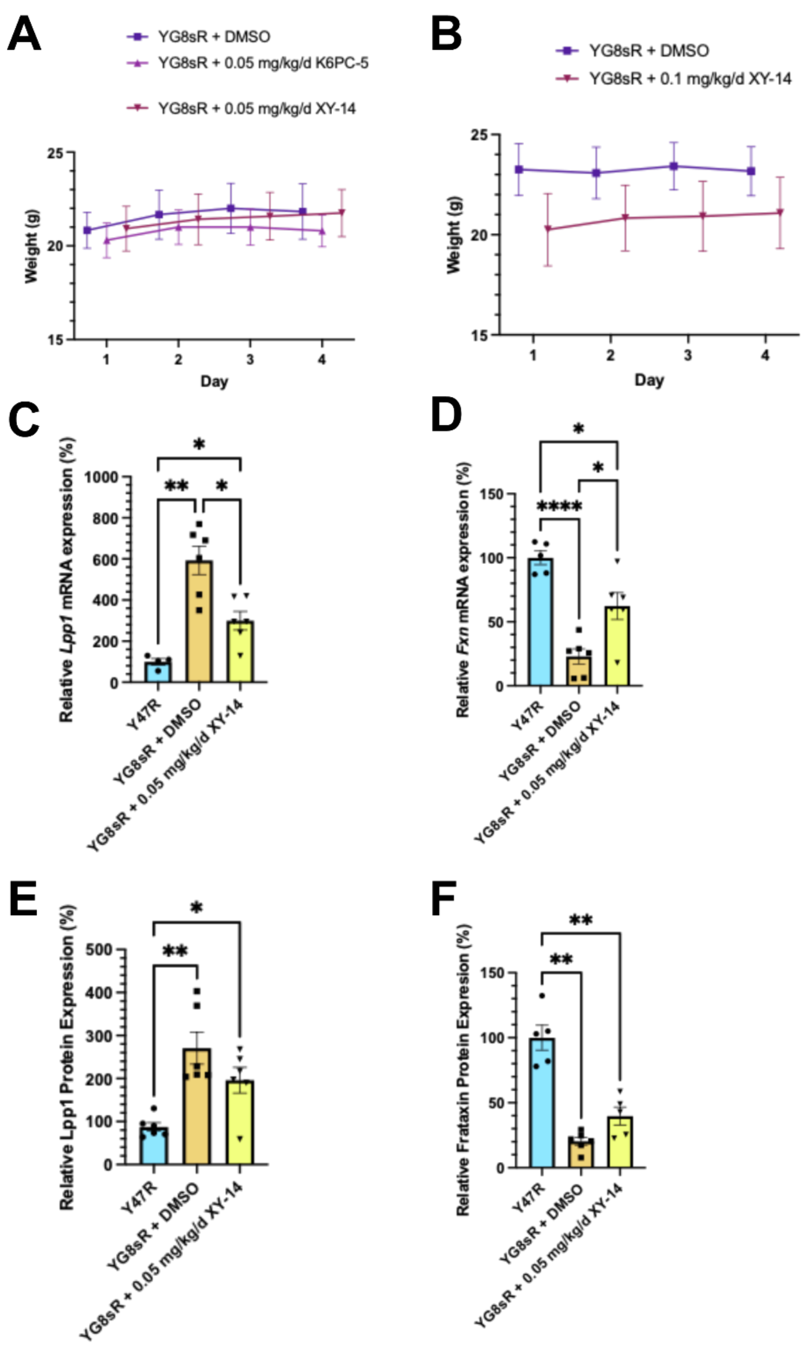

Supplementary Figure 6. Safety and efficacy of K6PC-5 and XY-14 in Y47R and YG8sR mouse models, and effects of XY-14 on Lpp and Fxn expression. Daily body weights (g) in YG8sR mice treated with (A) DMSO, 0.05 mg/kg/d K6PC-5, or 0.05 mg/kg/d XY-14, and (B) DMSO or 0.1 mg/kg/d XY-14, n=6. (C) *Lpp1* mRNA expression, measured by qRT-PCR, n=6, (D) Lpp1 protein expression, measured by ELISA, n=6, (E) *Fxn* mRNA expression, measured by qRT-PCR, n=6. (F) Frataxin expression, measured by ELISA, n=6-12. Statistical significance was determined using Welch's *t*-test or Welch's ANOVA with Dunnett's T3 multiple comparisons test (**P* <0.05, ***P* <0.01, ****P* <0.001, *****P* <0.0001).

Lpp1

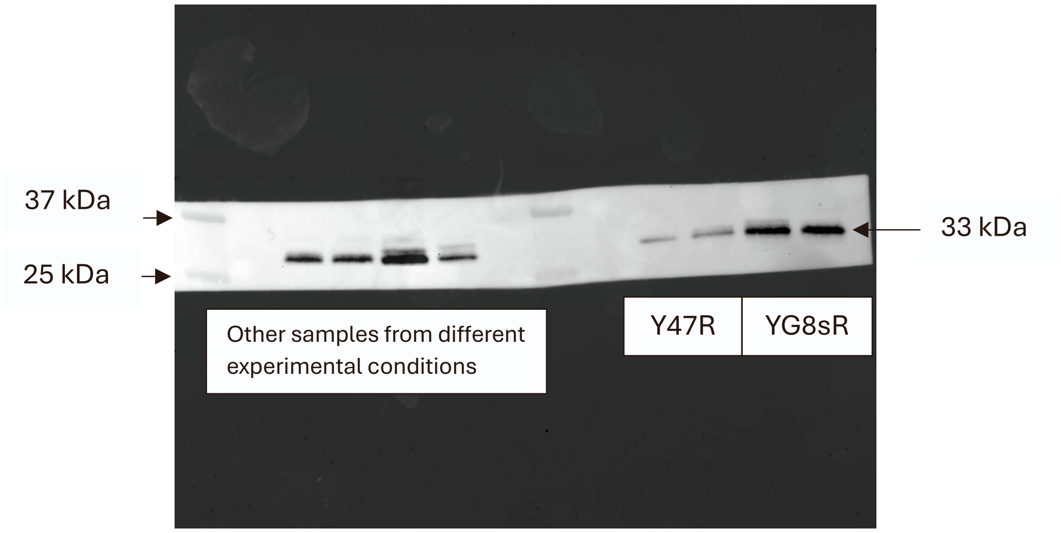

Sphk1

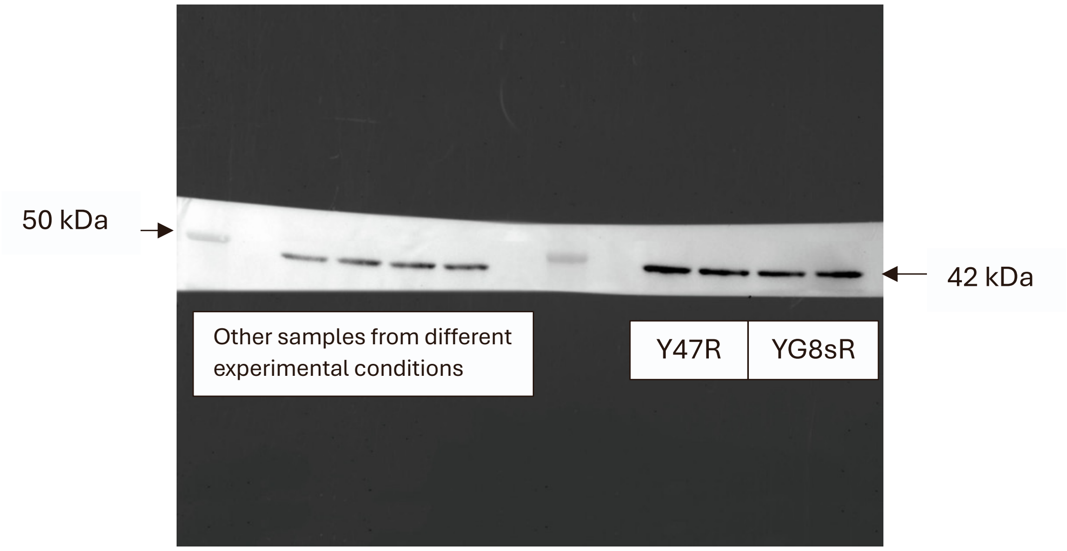

Frataxin

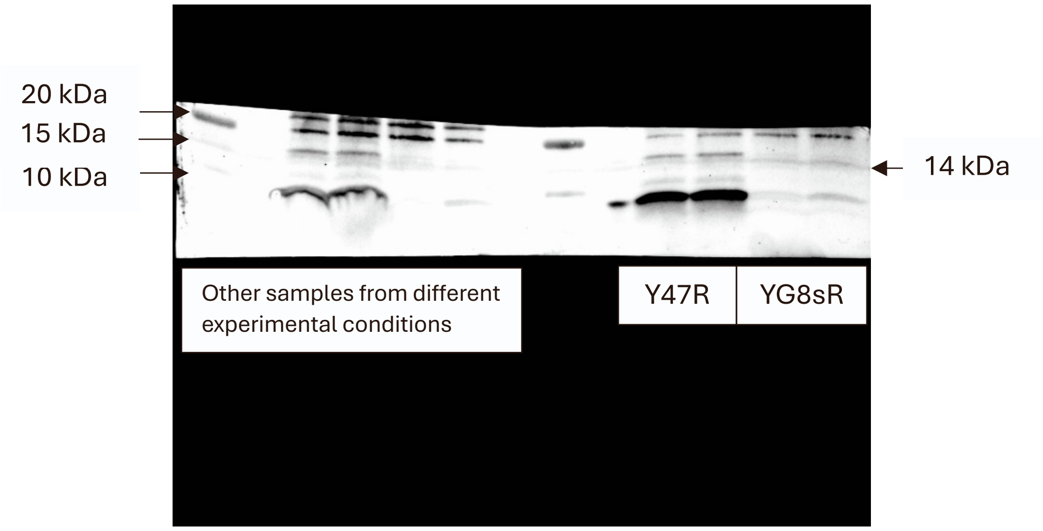

Tubulin

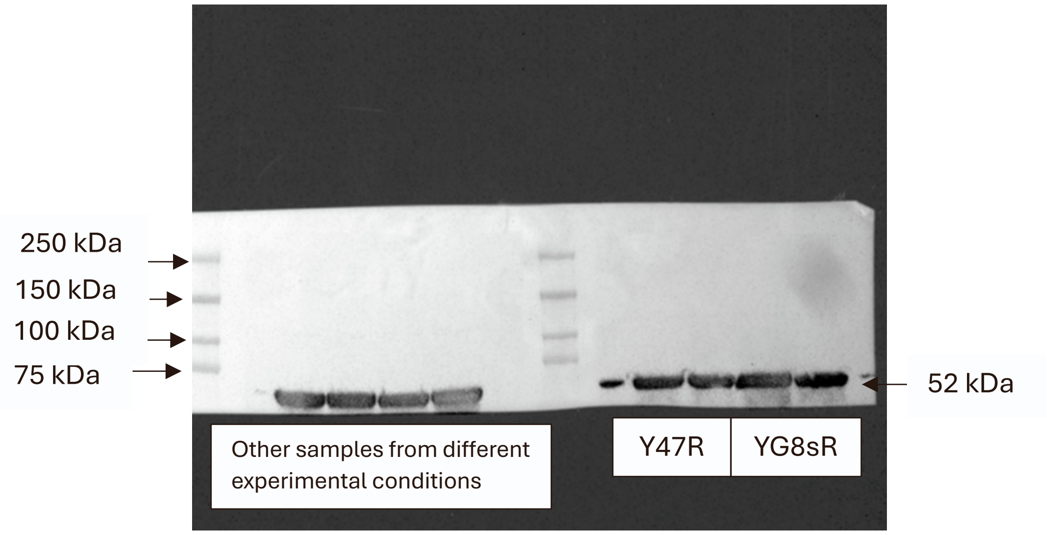

Data S1: Original Western blot images. Original, uncropped, unedited western blot images for Figure S1.
